# Isoniazid use, effectiveness, and safety for treatment of latent tuberculosis infection: a systematic review

**DOI:** 10.1590/0037-8682-0504-2023

**Published:** 2024-03-25

**Authors:** Bárbara Manuella Cardoso Sodré-Alves, Melina Mafra Toledo, Ivan Ricardo Zimmermann, Wildo Navegantes de Araújo, Noemia Urruth Leão Tavares

**Affiliations:** 1 Universidade de Brasília, Laboratório de Estudos Farmacêuticos, Brasília, DF, Brasil.; 2 Universidade de Brasília, Programa de Pós-Graduação em Ciências Farmacêuticas, Brasília, DF, Brasil.; 3 Universidade de Brasília, Programa de Pós-Graduação em Saúde Coletiva, Brasília, DF, Brasil.; 4 Universidade de Brasília, Programa de Pós-Graduação em Medicina Tropical, Brasília, DF, Brasil.; 5 Universidade de Brasília, Faculdade de Ceilândia, Brasília, DF, Brasil.; 6 Instituto Nacional de Ciência e Tecnologia para Avaliação de Tecnologias em Saúde, Porto Alegre, RS, Brasil.

**Keywords:** Latent tuberculosis infection, Isoniazid, Treatment adherence, Medication safety, Treatment effectiveness

## Abstract

**Background:**

The treatment strategy for latent tuberculosis infection is to reduce the number of tuberculosis cases and consequently reduce the transmission of pathogenic bacteria. This study aimed to determine the safety, effectiveness, and adherence of isoniazid use for latent tuberculosis infection treatment.

**Methods:**

To identify studies on isoniazid use for latent tuberculosis infection, five electronic databases were searched. The methods and results are presented in accordance with Preferred Reporting Items for Systematic Reviews and Meta-Analyses guidelines.

**Results:**

Most studies (53) used isoniazid for 9 months. The prevalence of use and adherence to treatment varied considerably (18% to 100%), and were evaluated by participant completion of isoniazid treatment for latent tuberculosis infection. The adverse events most frequently reported were hepatotoxicity, gastric intolerance, and neuropathy; the rates of occurrence ranged from < 1% to 48%. In the studies that evaluated the effectiveness of isoniazid for latent tuberculosis infection, the rate varied from 0 to 19.7% for patients who did not have active tuberculosis after the follow-up period.

**Conclusions:**

The importance of maintaining follow up for patients using isoniazid should be emphasized due to the risk of developing adverse events. Despite the treatment challenges, the rates of patients who used isoniazid and developed active tuberculosis during the follow-up period were low. We believe that isoniazid continues to contribute to tuberculosis control worldwide, and better care strategies are required.

## INTRODUCTION

Latent infection with *Mycobacterium tuberculosis* (LTBI) refers the moment when the individual is infected, and is characterized by a state of persistent immune response to stimulation by bacterial antigens with no clinical manifestations of tuberculosis (TB)^1^ ^-^ ^3^.

Within the context of the world strategy to end TB proposed by the World Health Organization (WHO), a reduction in the number of cases that evolve from LTBI to TB is one of several ways to achieve this objective^4^ ^-^ ^6^. Isoniazid (INH) is a medication used in TB preventive treatment (TPT) for individuals diagnosed with LTBI^7^ ^,^ ^8^. Patients who adhere to the treatment for the requisite period can have a 60% to 90% reduction in the risk for clinical manifestations and the potential to transmit the bacteria to their close contacts^9^.

The number of individuals undergoing preventive TB treatment has quadrupled since 2015 from 1 million in 2015 to > 4 million in 2019^5^ ^,^ ^10^ ^-^ ^12^. However, the coronavirus disease 2019 (COVID-19) pandemic in 2020 has had a significant impact on TB services. Data collected by the WHO from countries with a high TB burden demonstrated sharp drops in TB notifications in 2020 and, consequently, in LTBI screenings and preventive treatment^12^. Only 15.5 million people initiated TPT, 52% of the 5-year (2018-2022) target of 30 million^1^. This included 3.8 million people in 2022, which was above the pre-pandemic level of 3.6 million in 2019^1^.

However, the challenges in treating LTBI are not limited to the COVID-19 health emergency other factors influence the performance of programs to prevent the disease. Several studies have demonstrated how safety (addressing adverse reactions during treatment and drug interactions) and adherence to treatment by the user (who is not affected by symptoms but must use medication daily for months) can influence the efficacy of LTBI treatment^13^ ^-^ ^17^.

Understanding the global scenario and the data reported in scientific studies is important for identifying ways to help health managers and services improve TPT with INH and, in turn, promote a reduction in TB transmission rates. The present study aimed to determine the safety, effectiveness, and adherence to INH use for LTBI treatment as reported in scientific studies. 

## METHODS

This systematic review was conducted between January 2020 and March 2022 in accordance with the guidelines of the Preferred Reporting Items for Systematic Reviews and Meta-Analyses guidelines^18^. The study protocol was registered with PROSPERO^19^ under number CRD 42020176694.

### ● Research question

What is the use adherence, effectiveness, and safety of INH for the treatment of LTBI?

To explain the clinical issue, the eligibility criteria and the research strategy were based on the PI(E)CO (population, intervention [exposure], comparison, and outcome) elements, as follows: population, LTBI; exposure, INH; comparison, not applicable; and outcome, safety, effectiveness and adherence^20^.

### ● Data sources and search strategy

A comprehensive literature search of the Cochrane Central Register of Controlled Trials (CENTRAL), PubMed/Medline, Embase, Literatura Latino-Americana e do Caribe em Ciências da Saúde (LILACS), Scopus, and Web of Science databases was performed for articles published from inception to March 2020. Articles were searched using Medical Subject Headings (MeSH) descriptors and other non-standard descriptors, including: “Latent Tuberculosis” and “Isoniazid.” The descriptors were adapted for each database and combined using the Boolean operators “OR” and “AND.” The words used in the search are listed in Supplementary Table 1 The terms searched for the prevalence of INH use for LTBI.

### ● Inclusion and exclusion criteria

Descriptive and analytical observational studies that fulfilled the following criteria were included: use of INH for LTBI; determined rate of use of INH; published in English, Portuguese, or Spanish; and published from inception to March 2020.

Theoretical studies, systematic reviews, case reports, congress abstracts, letters to the editor, results, and award reports were excluded. Studies that addressed INH use only in pediatric patients, had methodological limitations that precluded analysis for the type and frequency of adverse reactions, and did not report an abstract or full text were also excluded. Studies indexed in ≥ 2 databases (duplicated) were considered only once.

### ● Study selection

After searching the databases, the selection process was performed in four stages: exclusion of pairwise and independent studies, analysis of article titles, evaluation of abstracts, full-text review of articles whose abstracts were selected, and manual screening of the references of the articles included after reading them in full. For stages 1 to 4, the studies were independently selected by two evaluators (BMCSA and MMT) using the Rayyan website^21^; for disagreements, a third evaluator analyzed and adjudicated the discrepancies. The overall degree of agreement between the evaluators at all stages was measured according to Rayyan and Cohen’s kappa (κ) index^22^. 

### ● Data extraction

After article selection, the following data were extracted: author(s), year of publication, journal, study location, study type, study setting, study duration, type of sample selection, sample size, study limitations, clinical findings, rate of INH use, method for identifying adverse reactions, type of adverse reactions, severity of adverse reactions, rates of adverse reactions, management of adverse reactions, assessment of therapeutic responses, and other pertinent observations.

### ● Quality assessment

*Evaluation of study quality -* Quality assessment of the included observational cohort and cross-sectional studies was performed using the Quality Assessment Tool for Observational Cohort and Cross-Sectional Studies, for which 14 specific questions were answered with “Yes,” “No” and “Not applicable”^23^. After analyzing the instrument responses, the evaluators classified the study quality as good, fair, or poor, and comments on the decisions were documented in the latter cases. All quality assessments of the studies included in this systematic review were performed by two independent reviewers (BMCSA and MMT), and discrepancies were resolved by the consensus decision-making process.

## RESULTS

**FIGURE 1:**
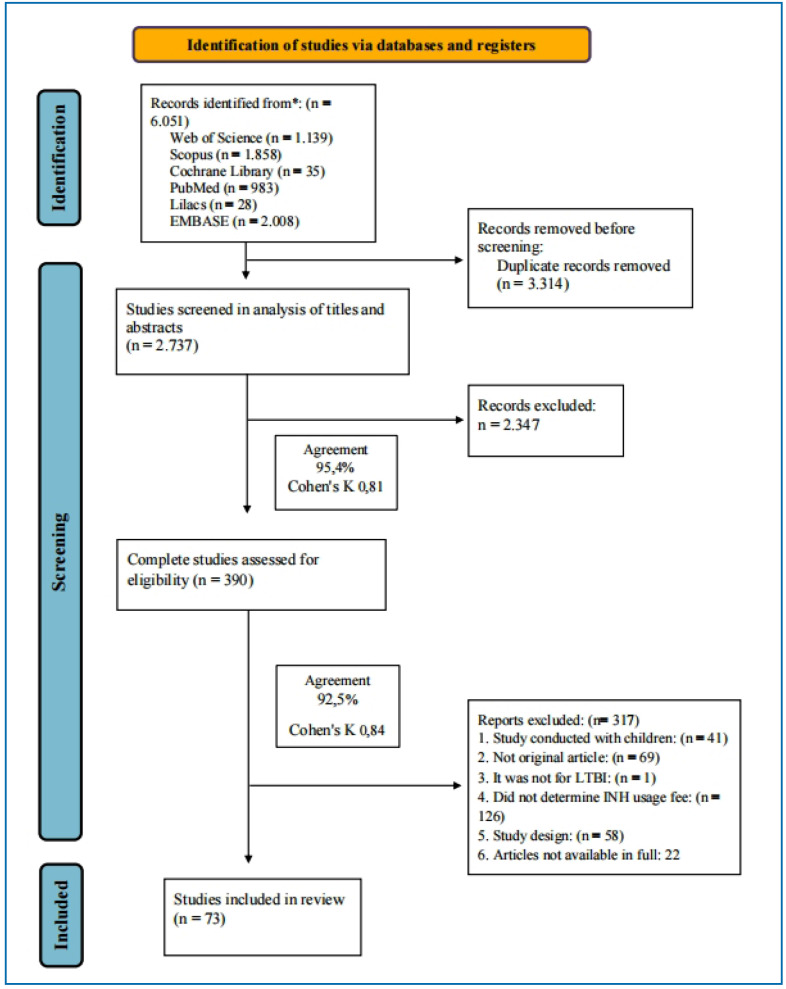
Study selection flowchart. **INH:** isoniazid; **LTBI:** latent tuberculosis infection.

A total of 6051 potentially relevant studies were retrieved, 73 of which were included in this systematic review (Figure 1 and Supplementary Table 2)^24^ ^-^ ^95^. In the title and abstract evaluation stage, there was virtually perfect agreement (95.4% [2611/2737]) between the articles; Cohen’s κ, 0.81), and in the evaluation of the full texts, there was virtually perfect agreement (92.5 % [347/390]; Cohen's κ: 0.84) between the evaluators.

### ● Study characteristics

**FIGURE 2:**
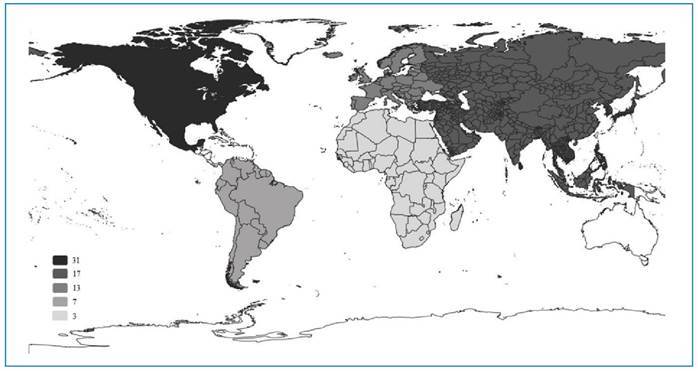
Number of studies included, by continent.

The studies included in this systematic review were conducted across countries in five continents (Figure 2). Three of the included studies did not report where they were conducted^54^ ^,^ ^62^ ^,^ ^88^. The most frequent settings were: outpatient clinics and clinics (n = 41)^24^ ^-^ ^26^ ^,^ ^30^ ^,^ ^35^ ^-^ ^38^ ^,^ ^40^ ^-^ ^43^ ^,^ ^46^ ^-^ ^52^ ^,^ ^56^ ^-^ ^58^ ^,^ ^61^ ^,^ ^67^ ^,^ ^68^ ^,^ ^71^ ^-^ ^73^ ^,^ ^75^ ^,^ ^81^ ^,^ ^82^ ^,^ ^84^ ^,^ ^85^ ^,^ ^87^ ^,^ ^89^ ^-^ ^95^; hospitals (n =19)^24^ ^,^ ^28^ ^,^ ^39^ ^,^ ^40^ ^,^ ^45^ ^,^ ^53^ ^,^ ^55^ ^,^ ^59^ ^,^ ^60^ ^,^ ^64^ ^,^ ^66^ ^,^ ^70^ ^,^ ^74^ ^,^ ^76^ ^,^ ^77^ ^,^ ^80^ ^,^ ^78^ ^,^ ^79^ ^,^ ^83^; and penitentiaries (n = 4)^29^ ^,^ ^31^ ^,^ ^33^ ^,^ ^63^.

Most of the included studies (n = 49) did not specify patient profiles^24^ ^-^ ^33^ ^,^ ^35^ ^-^ ^37^ ^,^ ^40^ ^-^ ^44^ ^,^ ^47^ ^,^ ^49^ ^,^ ^51^ ^,^ ^52^ ^,^ ^54^ ^,^ ^55^ ^,^ ^57^ ^-^ ^59^ ^,^ ^60^ ^,^ ^63^ ^-^ ^65^ ^,^ ^67^ ^,^ ^68^ ^,^ ^70^ ^,^ ^71^ ^,^ ^77^ ^,^ ^79^ ^,^ ^81^ ^-^ ^87^ ^,^ ^89^ ^,^ ^90^ ^,^ ^93^ ^,^ ^94^, four studies involved patients with inflammatory bowel disease or immune-mediated inflammatory diseases^72^ ^,^ ^73^ ^,^ ^76^ ^,^ ^80^, four involved human immunodeficiency virus (HIV)-positive adults^56^ ^,^ ^61^ ^,^ ^74^ ^,^ ^91^, four involved patients who transplanted or had transplants ^34^ ^,^ ^50^ ^,^ ^62^ ^,^ ^66^, and six involved health professionals^38^ ^,^ ^39^ ^,^ ^45^ ^,^ ^48^ ^,^ ^78^ ^,^ ^88^.

### ● Medications used

Regarding the various treatment alternatives for LTBI globally, among the studies analyzed, 53 used INH for 9 months^24^ ^-^ ^28^ ^,^ ^30^ ^-^ ^34^ ^,^ ^38^ ^-^ ^46^ ^,^ ^49^ ^-^ ^54^ ^,^ ^58^ ^,^ ^61^ ^,^ ^63^ ^,^ ^64^ ^,^ ^67^ ^,^ ^69^ ^,^ ^72^ ^-^ ^74^ ^,^ ^76^ ^,^ ^77^ ^-^ ^79^ ^,^ ^80^ ^,^ ^81^ ^,^ ^82^ ^-^ ^87^ ^,^ ^89^ ^,^ ^91^ ^,^ ^92^ ^,^ ^94^ ^,^ ^95^, 40 used INH for 6 months^24^ ^,^ ^26^ ^,^ ^27^ ^,^ ^29^ ^,^ ^34^ ^-^ ^36^ ^,^ ^39^ ^,^ ^42^ ^-^ ^44^ ^,^ ^47^ ^-^ ^49^ ^,^ ^53^ ^,^ ^55^ ^,^ ^56^ ^,^ ^57^ ^,^ ^59^ ^-^ ^62^ ^,^ ^64^ ^,^ ^65^ ^,^ ^67^ ^,^ ^68^ ^,^ ^70^ ^,^ ^71^ ^,^ ^72^ ^,^ ^74^ ^,^ ^75^ ^,^ ^84^ ^,^ ^86^ ^-^ ^90^ ^,^ ^93^ ^,^ ^94^ ^,^ ^95^, and two used INH for 12 months^66^. Rifampicin monotherapy for 4 months was investigated in 28 studies^25^ ^-^ ^28^ ^,^ ^32^ ^,^ ^35^ ^,^ ^37^ ^,^ ^40^ ^,^ ^41^ ^,^ ^43^ ^,^ ^44^ ^,^ ^45^ ^,^ ^48^ ^,^ ^51^ ^,^ ^52^ ^,^ ^53^ ^,^ ^54^ ^,^ ^58^ ^,^ ^63^ ^,^ ^64^ ^,^ ^65^ ^,^ ^69^ ^,^ ^71^ ^,^ ^76^ ^-^ ^78^ ^,^ ^81^ ^,^ ^90^ ^,^ ^78^ (Supplementary Table 2).

Among the combination therapies for LTBI, 11 studies used INH + rifampicin ^49^ ^,^ ^63^ ^,^ ^71^ ^,^ ^74^ ^-^ ^76^ ^,^ ^78^ ^,^ ^79^ ^,^ ^81^ ^,^ ^88^ ^,^ ^90^, 10 used INH + rifapentine^28^ ^,^ ^30^ ^,^ ^31^ ^,^ ^33^ ^,^ ^42^ ^,^ ^45^ ^,^ ^49^ ^,^ ^50^ ^,^ ^52^ ^,^ ^83^, eight used rifampicin + pyrazinamide^24^ ^,^ ^29^ ^,^ ^43^ ^,^ ^47^ ^,^ ^51^ ^,^ ^63^ ^,^ ^68^ ^,^ ^89^, and three used rifampicin + INH + pyrazinamide + ethambutol^46^ ^,^ ^49^ ^,^ ^74^. The drug combinations that were used less frequently for TB prophylaxis were rifampicin + INH^24^ ^,^ ^40^ ^,^ ^67^, rifampicin + ethambutol ^70^, and rifampicin + INH + pyrazinamide ^24^ ^,^ ^68^ ^,^ ^74^.

### ● INH use prevalence

Five studies that fulfilled the inclusion criteria reported separately the number and proportion of individuals who used INH for 6 and 9 months: 51 (41.8%) for 6 months, 26 (21.3%) for 9 months, and 22 (18.0%) for 6 months^24^; 181 (9.1%) for 6 months and 1674 (84.0%) for 9 months^43^; 7332 (54.9%) for 6 months, 4298 (32.2%) for 9 months; 263 (2%) for ≤ 4 months^67^; 466 (77.8%) for 6 months and 80 (13.4%) for 9 months^74^; 1 (4%) for 6 months, and 3 (13%) for 9 months^53^.

A large variation in INH use prevalence was observed in studies that did not distinguish the duration of treatment: 68 studies reported 0.3% to 98.6% of the study participants used INH for LTBI; 15 (22.1%) had prevalence within the range of the 1st quartile, 13 (19.1%) in the 2nd quartile, 17 (25.0%) in the 3rd quartile, and 23 (33.8%) in the 4th quartile^25^ ^-^ ^42^ ^,^ ^44^ ^-^ ^52^ ^,^ ^54^ ^-^ ^66^ ^,^ ^68^ ^-^ ^73^ ^,^ ^75^ ^-^ ^95^ (Supplementary Table 3).

### ● Treatment adherence

**FIGURE 3:**
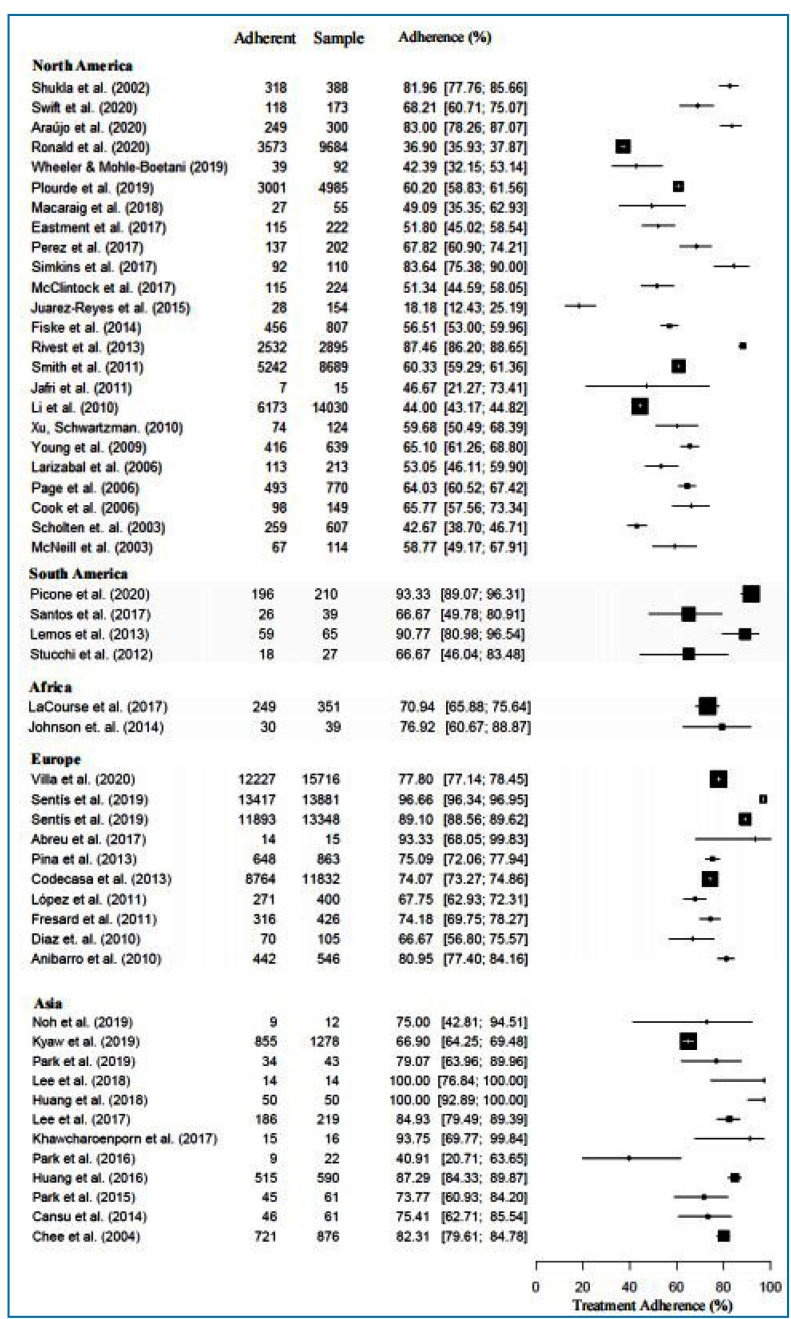
Treatment adherence in continents.

In the 52 studies that similarly reported measurements for adherence to treatment with INH, a large variation was observed, both in the number of participants-ranging from 5 to > 12,000 individuals-and in the adherence rate, which ranged from 18% to 100% of study participants completing INH treatment for LTBI. The studies conducted in Europe had were more consistent adherence estimates with similar magnitudes when compared to other continents (Figure 3 and Supplementary Table 3).

One (2.0%) study reported an adherence rate within the range of the 1st quartile, 10 (19.2%) in the 2nd quartile, 25 (48.1%) in the 3rd quartile and 16 (30.8%) in the 4th quartile^25^ ^-^ ^27^ ^,^ ^29^ ^-^ ^31^ ^,^ ^33^ ^-^ ^38^ ^,^ ^40^ ^-^ ^42^ ^,^ ^46^ ^-^ ^52^ ^,^ ^54^ ^-^ ^61^ ^,^ ^63^ ^-^ ^65^ ^,^ ^67^ ^,^ ^70^ ^-^ ^73^ ^,^ ^77^ ^-^ ^79^ ^,^ ^80^ ^-^ ^84^ ^,^ ^87^ ^,^ ^88^ ^,^ ^90^ ^,^ ^91^ ^,^ ^95^.

Eight studies reported that INH treatment adherence rates differed from those in most studies, such as individuals who purchased doses, received 180 doses over the 7-month period, were compliant with therapy, completed < 6 months of treatment, completed 6 months and failed to complete the final 3 months^24^ ^,^ ^32^ ^,^ ^39^ ^,^ ^43^ ^,^ ^44^ ^,^ ^45^ ^,^ ^62^ ^,^ ^94^. Twelve studies did not assess adherence to treatment with INH^28^ ^,^ ^53^ ^,^ ^66^ ^,^ ^68^ ^,^ ^69^ ^,^ ^74^ ^,^ ^75^ ^,^ ^85^ ^,^ ^86^ ^,^ ^89^ ^,^ ^92^ ^,^ ^93^.

### ● INH adverse events

An analysis of the types and frequencies of adverse events associated with INH was heterogeneous among the studies included in this review. In 38 studies, the most frequent events reported were hepatotoxicity, gastric intolerance, and neuropathy, the occurrence rates of adverse events from INH, ranged from < 1% to 48%^24^ ^,^ ^25^ ^,^ ^26^ ^,^ ^30^ ^,^ ^31^ ^,^ ^33^ ^,^ ^34^ ^,^ ^37^ ^,^ ^40^ ^,^ ^41^ ^,^ ^42^ ^,^ ^45^ ^,^ ^47^ ^,^ ^50^ ^,^ ^51^ ^,^ ^54^ ^,^ ^56^ ^-^ ^58^ ^,^ ^62^ ^,^ ^63^ ^,^ ^64^ ^-^ ^66^ ^,^ ^68^ ^,^ ^71^ ^,^ ^73^ ^,^ ^76^ ^-^ ^79^ ^,^ ^81^ ^-^ ^83^ ^,^ ^86^ ^,^ ^88^ ^,^ ^89^ ^,^ ^91^ ^,^ ^95^ (Supplementary Table 4). Two studies analyzed but did not identify adverse events among the participants^55^ ^,^ ^60^.

The occurrence of adverse events from INH was not analyzed in 32 studies^27^ ^-^ ^29^ ^,^ ^32^ ^,^ ^35^ ^,^ ^36^ ^,^ ^38^ ^,^ ^39^ ^,^ ^43^ ^,^ ^44^ ^,^ ^46^ ^,^ ^48^ ^,^ ^49^ ^,^ ^52^ ^,^ ^53^ ^,^ ^59^ ^,^ ^61^ ^,^ ^67^ ^,^ ^69^ ^,^ ^70^ ^,^ ^72^ ^,^ ^74^ ^,^ ^75^ ^,^ ^77^ ^,^ ^80^ ^,^ ^84^ ^,^ ^85^ ^,^ ^87^ ^,^ ^90^ ^,^ ^92^ ^-^ ^94^.

### ● Treatment effectiveness

The effectiveness of INH treatment for LTBI was measured by a diagnosis of active TB; heterogeneity was observed in the follow-up period of the participants to identify this outcome; some researchers followed up only during INH use, others continued to follow up the participants after the end of treatment or used infectious disease reporting systems to identify TB activation in those who used INH for LTBI.

Regarding the assessment of active TB development during and/or after a follow-up period after INH use for LTBI, 20 studies reported a variation from < 1% to 19.7%, with the highest rate among HIV-positive individuals who evolved to active TB after preventive therapy. In other participants, the rates did not exceed 10%, and most remained below 5%^28^ ^,^ ^36^ ^,^ ^37^ ^,^ ^49^ ^,^ ^56^ ^,^ ^57^ ^,^ ^61^ ^-^ ^63^ ^,^ ^71^ ^,^ ^75^ ^,^ ^76^ ^,^ ^79^ ^,^ ^80^ ^,^ ^83^ ^,^ ^85^ ^-^ ^87^ ^,^ ^89^ ^,^ ^93^. Another 17 studies evaluated the effectiveness of INH treatment and did not identify active TB after the follow-up period^30^ ^,^ ^34^ ^,^ ^41^ ^,^ ^46^ ^,^ ^48^ ^,^ ^50^ ^,^ ^53^ ^,^ ^55^ ^,^ ^60^ ^,^ ^66^ ^,^ ^72^ ^,^ ^73^ ^,^ ^84^ ^,^ ^88^ ^,^ ^91^ ^,^ ^92^ ^,^ ^94^ (Supplementary Table 5).

Thirty-five studies did not assess INH effectiveness as a preventive therapy for TB with INH^24^ ^,^ ^26^ ^-^ ^27^ ^,^ ^29^ ^,^ ^31^ ^-^ ^33^ ^,^ ^35^ ^,^ ^38^ ^-^ ^40^ ^,^ ^42^ ^-^ ^45^ ^,^ ^47^ ^,^ ^51^ ^,^ ^52^ ^,^ ^54^ ^,^ ^58^ ^,^ ^59^ ^,^ ^64^ ^,^ ^65^ ^,^ ^67^ ^-^ ^70^ ^,^ ^74^ ^,^ ^77^ ^,^ ^78^ ^,^ ^81^ ^,^ ^82^ ^,^ ^90^ ^,^ ^95^ ^,^ ^90^.

### ● Methodological quality analysis

**FIGURE 4:**
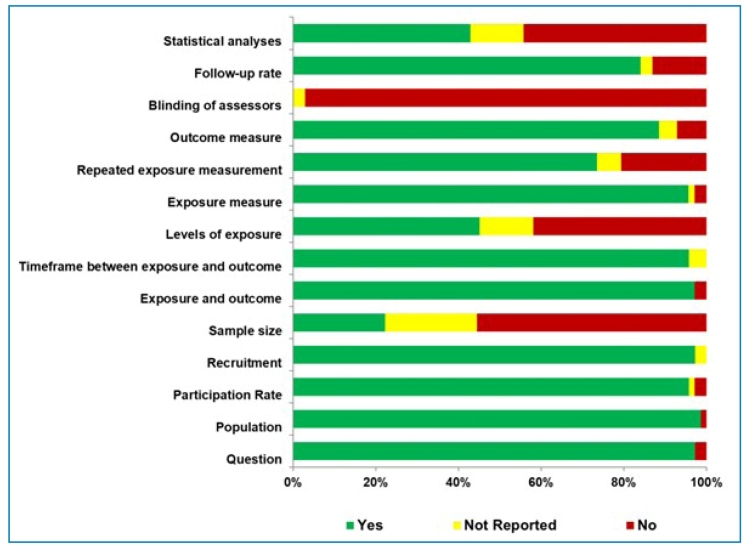
Quality assessment of cross-sectional studies.

In general, the studies included in this systematic review demonstrated fair methodological quality. Thirty-six studies were considered to have good methodological quality^25^ ^-^ ^28^ ^,^ ^32^ ^,^ ^34^ ^,^ ^39^ ^,^ ^41^ ^,^ ^42^ ^-^ ^44^ ^,^ ^46^ ^,^ ^47^ ^,^ ^49^ ^,^ ^50^ ^,^ ^52^ ^,^ ^54^ ^,^ ^58^ ^,^ ^61^ ^,^ ^62^ ^,^ ^64^ ^,^ ^66^ ^-^ ^68^ ^,^ ^70^ ^,^ ^74^ ^,^ ^77^ ^,^ ^79^ ^,^ ^80^ ^,^ ^81^ ^,^ ^83^ ^,^ ^87^ ^,^ ^88^ ^,^ ^93^ and 37 had “regular” quality^24^ ^,^ ^29^ ^-^ ^31^ ^,^ ^33^ ^,^ ^35^ ^-^ ^38^ ^,^ ^40^ ^,^ ^45^ ^,^ ^48^ ^,^ ^51^ ^,^ ^53^ ^,^ ^55^ ^-^ ^57^ ^,^ ^59^ ^,^ ^60^ ^,^ ^63^ ^,^ ^65^ ^,^ ^69^ ^,^ ^71^ ^-^ ^73^ ^,^ ^75^ ^,^ ^76^ ^,^ ^78^ ^,^ ^82^ ^,^ ^84^ ^-^ ^86^ ^,^ ^89^ ^,^ ^90^ ^-^ ^92^ ^,^ ^94^ ^,^ ^95^. The detailed results of the quality assessment using the Quality Assessment Tool for Observational Cohort and Cross-Sectional Studies for the 73 studies included in this systematic review are summarized in Figure 4 and Supplementary Table 6.

## DISCUSSION

Although the efficacy of INH for TPT was first demonstrated > 60 years ago in randomized controlled trials conducted by the United States Health Services^96^ ^,^ ^97^ ^,^ ^98^ ^,^ ^99^, to our knowledge, this was the first systematic review to investigate the use of INH for LTBI treatment and its safety, adherence, and effectiveness.

Rifapentine has been incorporated into the Brazilian Unified Health System and adopted within TPT strategies in other countries, and INH continues to be a recommended medication, both as monotherapy and in combination with rifapentine. Therefore, in the context of daily life, the safety, adherence, and effectiveness of this low-cost medicine continue to be relevant for health managers and professionals. In addition, it is worth highlighting the temporality impact of this review because the searches were concentrated in a period before the incorporation of the new TPT scheme.

LTBI treatment and systematic testing are strongly recommended in the guidelines for LTBI management worldwide, with evidence promoting an annual reduction of up to 10% in TB morbidity^6^ ^,^ ^100^. The profiles of patients using INH for LTBI reflect these guidelines, in which INH was predominantly administered to individuals at greater risk for developing the active disease such as those living with HIV, adult and child contacts of patients with pulmonary TB, patients initiating anti-tumor necrosis factor treatment, patients undergoing dialysis, and those preparing for transplantation^6^ ^,^ ^101^.

The most studied outcomes were adverse events resulting from medication use and treatment adherence; fewer studies investigated the effectiveness of INH treatment as a preventive action against active TB in the medium and long term. All these aspects of treatment are relevant to the control and eradication of active TB worldwide; as such, it is important to determine the period during which individuals remain without risk for developing active TB.

The prevalence of INH use varied considerably among studies. As monotherapy with INH for 9 months was the most frequent, other anti-TB drugs, such as R and/or P, were also analyzed. Globally, INH regimens for 6 or 9 months are options; however, despite their proven efficacy, they carry a higher risk of toxicity and lower treatment completion rates, which reduces their efficacy. Nevertheless, there is a consensus that a 9-month regimen of INH therapy has been adopted as the standard comparator to assess shorter-course schedules^102^.

Adherence to INH treatment among the study participants varied greatly and was measured in most studies by treatment completion. This variation in the proportion of individuals who completed treatment for LTBI has also been reported in other systematic reviews^13^ ^,^ ^103^. The most important factor for this treatment is the number of doses, not merely the duration of the treatment chosen^8^.

Although the importance for completion of LTBI treatment to control active TB globally is well-established in the literature and health services, adherence levels at treatment initiation and completion have been consistently suboptimal^104^ ^,^ ^105^. Strategies have been adopted to increase treatment adherence rates such as reminder telephone calls before appointments, reminder cards delivered at the first appointment, and nursing home visits for participants who cannot physically attend appointments (s)^106^. 

Health professionals should seek strategies that best meet the profiles of patients treated with their service because adherence is a multifactorial activity. However, there is evidence that therapy-(treatment regimen) and disease-(duration) related factors have little or no impact on adherence^107^.

It is important to emphasize that researchers and health professionals who want to measure treatment adherence, whether for LTBI or others, are clear about the existing methods and make use of them in their studies and/or health services and opt for those methods most consider reliable and practical. 

We acknowledge that there is no perfect method and that multimeasure adhesion approaches may be the best solutions. In this context, approaches or methods for measuring treatment adherence can be both subjective and objective, including direct measures such as secondary database analysis, electronic medication packaging devices, pill counting, patient assessments, and self-reports. Subjective approaches often provide explanations for patient nonadherence, whereas objective measures contribute to a more accurate recording of a patient's behavior while taking medication^108^.

The adverse events reported in these studies corroborate with those predicted in the literature and in the drug leaflet (product monograph); less than half of the study participants mainly experienced hepatotoxicity and gastric intolerance^109^. A study from the Republic of Korea that analyzed data from a surveillance system for adverse events reported that among the anti-TB drugs, INH was the second most common in causing adverse events in patients (24%), with R being the first (28.7%). This systematic review, identified the most common adverse events were in the gastrointestinal system disorders (32.0%), followed by skin and the limbs (25.9%) and the liver and biliary system (14.2%)^110^.

However, Campbell et al.^111^ analyzed phase 2 clinical trials and reported that a 4-month regimen of R resulted in approximately half of the INH adverse events during 9 months. Thus, the importance of surveillance and monitoring of treatment after the drug testing phases (phases 2 and 3 clinical trials) is noted for monitoring outcomes of the real-world level of treatment, especially those with evidence of a greater chance for causing adverse events.

The effectiveness of preventive active TB treatment can, be based on the screening of new TB cases in a population sample that underwent treatment for LTBI^103^. Evaluation of INH treatment effectiveness was the outcome of less interest in the studies included in this review, which may be explained by the high cost of developing studies that conduct long-term participant follow-up and the need for laboratory screening over time to identify active disease. 

Some of the included studies used approaches that made it possible to identify the relationship between the effectiveness and use of the drug because they evaluated both the activation of TB during INH use and followed the participants for a prolonged period after the completion or abandonment of treatment; thus it was possible to correlate treatment completion and active TB development. This type of assessment has positive potential in TB control and may be incorporated into health services as a treatment assessment tool and a quick-action measure in patients who may experience activation of *M. tuberculosis* bacterium^112^.

Although studies in three languages were included, a limitation of this systematic review was language bias due to the non-inclusion of studies published in other languages from countries with a high burden of TB infection, such as studies published in French, Russian, Hindi, and Mandarin^113^. Some articles (22) were not included because they required payment for access, and we had limited resources, or because they were not available in full, which could have impacted the results presented in this review. Adherence, safety, and effectiveness were either underestimated or overestimated. Another limitation was the methodological heterogeneity in identifying and reporting results, which limited direct comparisons between studies and, consequently, meta-analyses of the data. 

Thus, we encourage future researchers to adopt clear and widely accepted definitions in the literature for treatment adherence and adverse events as well as the implementation of validated instruments to identify these outcomes, such as the Naranjo algorithm^114^ ^,^ ^115^. The prevalence, use, and outcomes of other drugs that were not used as descriptors for the search strategy may have been underestimated. 

Modeling studies have suggested that without linking the diagnosis and treatment of both active TB and LTBI, it will not be possible to achieve the targets for reducing TB cases by 2025 or its elimination by 2050^116^. The results of this review may help the scientific community improve methods and outcomes of interest for future evaluations of INH treatment for LTBI, as well as for health managers to understand the high use, safety, adherence, and effectiveness of INH treatment, its positive impacts, and the difficulties that need to be overcome for TB preventive treatment.

Understanding the treatment for LTBI is important to identify ways that can help health mangers and services, and the general population improve prophylactic treatment with INH and consequently promote a reduction in TB transmission rates. Our findings indicated that INH has been widely used in the world as a prophylactic treatment for TB, with INH adherence rates > 50%. It is important to emphasize the importance of maintaining follow-up of patients who use INH because of the risk of developing adverse events from the drug. Despite the treatment challenges, we identified low rates of patients who used INH and developed active TB during the follow-up period. We believe that INH continues to contribute to TB control worldwide. The included studies demonstrated good prevention rates for active TB and that better care is needed, such as expanding access to treatments with safe regimens, improving therapeutic convenience for the patients, and more structured, frequent monitoring services for these patients.

## References

[1] World Health Organization (WHO) (2023). Global tuberculosis report 2023.

[2] Busatto C, Reis AJ, Valim ADM, Nunes LDS., Carneiro M, Possuelo LG (2015). Tuberculose ativa versus Tuberculose Latente: uma revisão de literatura. J Infect Control.

[3] World Health Organization (WHO) (2017). Global tuberculosis report 2017.

[4] Br Bloom, Atun R (2016). Back to the future: Rethinking global control of tuberculosis. Sci Transl Med.

[5] Harding E (2020). WHO global progress report on tuberculosis elimination. Lancet Respir Med.

[6] World Health Organization (WHO) (2015). Guidelines on the management of latent tuberculosis infection.

[7] Getahun H, Matteelli A, Abubakar I, Aziz MA, Baddeley A, Barreira D (2015). Management of latent Mycobacterium tuberculosis infection: WHO guidelines for low tuberculosis burden countries. Eur Respir J.

[8] Ministério da Saúde (MS). Secretaria de Vigilância em Saúde (2019). Manual de Recomendações para o Controle da Tuberculose no Brasil.

[9] Ai JW, Ruan QL, Liu QH, Zhang WH (2016). Updates on the risk factors for latent tuberculosis reactivation and their managements. Emerg Microbes Infect.

[10] Ross JM, Badje A, Rangaka MX, Walker AS, Shapiro AE, Thomas KK (2021). Isoniazid preventive therapy plus antiretroviral therapy for the prevention of tuberculosis: a systematic review and meta-analysis of individual participant data. Lancet HIV.

[11] Hsieh YL, Jahn A, Menzies NA, Yaesoubi R, Salomon JA, Girma B (2020). Evaluation of 6-Month Versus Continuous Isoniazid Preventive Therapy for Mycobacterium tuberculosis in Adults Living With HIV/AIDS in Malawi. J Acquir Immune Defic Syndr.

[12] World Health Organization (WHO) (2020). Global tuberculosis report 2020.

[13] Liu Y, Birch S, Newbold KB, Essue BM (2018). Barriers to treatment adherence for individuals with latent tuberculosis infection: A systematic search and narrative synthesis of the literature. Int J Health Plann Manage.

[14] Pease C, Hutton B, Yazdi F, Wolfe D, Hamel C, Quach P (2017). Efficacy and completion rates of rifapentine and isoniazid (3HP) compared to other treatment regimens for latent tuberculosis infection: a systematic review with network meta-analyses. BMC Infect Dis.

[15] Pease C, Hutton B, Yazdi F, Wolfe D, Hamel C, Barbeau P (2018). A systematic review of adverse events of rifapentine and isoniazid compared to other treatments for latent tuberculosis infection. Pharmacoepidemiol Drug Saf.

[16] Sandgren A, Vonk Noordegraaf-Schouten M, van Kessel F, Stuurman A, Oordt-Speets A, van der Werf MJ (2016). Initiation and completion rates for latent tuberculosis infection treatment: a systematic review. BMC Infect Dis.

[17] Stuurman AL, Vonk Noordegraaf-Schouten M, van Kessel F, Oordt-Speets AM, Sandgren A, van der Werf MJ (2016). Interventions for improving adherence to treatment for latent tuberculosis infection: a systematic review. BMC Infect Dis.

[18] Matthew JP Joanne EMK, Patrick M B Isabelle B, Tammy CH Cynthia DM (2021). The PRISMA 2020 statement: an updated guideline for reporting systematic reviews. BMJ.

[19] Booth A, Clarke M, Dooley G, Ghersi D, Moher D, Petticrew M (2013). PROSPERO at one year: an evaluation of its utility. Systematic Reviews.

[20] Cooke A, Smith D, Booth A (2012). Beyond PICO: The SPIDER tool for qualitative evidence synthesis. Qual Health Res.

[21] Ouzzani M, Hammady H, Fedorowicz Z, Elmagarmid A (2016). Rayyan-a web and mobile app for systematic reviews. Systematic Reviews.

[22] Landis JR, Koch GG (1977). The Measurement of Observer Agreement for Categorical Data. Biometrics.

[23] National Heart, Lung, and Blood Institute (2021). Study Quality Assessment Tools. National Heart, Lung, and Blood Institute Washington.

[24] Diaz A, Diez M, Bleda MJ, Aldamiz M, Camafort M, Camino X (2010). Eligibility for and outcome of treatment of latent tuberculosis infection in a cohort of HIV-infected people in Spain. BMC Infect Dis.

[25] Young H, Wessolossky M, Ellis J, Kaminski M, Daly JS (2009). A Retrospective evaluation of completion rates, total cost, and adverse effects for treatment of latent tuberculosis infection in a public health clinic in central Massachusetts. Clin Infect Dis.

[26] Li J, Munsiff SS, Tarantino T, Dorsinville M (2010). Adherence to treatment of latent tuberculosis infection in a clinical population in New York City. Int J Infect Dis.

[27] Smith BM, Schwartzman K, Bartlett G, Menzies D (2011). Adverse events associated with treatment of latent tuberculosis in the general population. CMAJ.

[28] Flynn AG, Aiona K, Haas MK, Reves R, Belknap R (2020). Clinical characteristics of active tuberculosis diagnosed after starting treatment for latent tuberculosis infection. Clin Infect Dis.

[29] Lincoln T, Brannan GL, Lynch V, Conklin TJ, Clancey T, Rose DN (2004). Completing tuberculosis prophylaxis in jail: Targeting treatment and comparison of rifampin/pyrazinamide with isoniazid regimens. Int J Tuberc Lung Dis.

[30] Sweeney TL, Ahern JW, Alston KW (2017). Completion Rate and Safety of 12-Dose Isoniazid and Rifapentine for Latent Tuberculosis in a Predominantly Refugee Cohort. Infect Dis Clin Pract.

[31] Juarez-Reyes M, Gallivan M, Chyorny A, O'Keeffe L, Shah NS (2016). Completion rate and side-effect profile of three-month isoniazid and rifapentine treatment for latent tuberculosis infection in an Urban county jail. Open Forum Infect Dis.

[32] Rivest P, Street MC, Allard R (2013). Completion rates of treatment for latent tuberculosis infection in Quebec, Canada From 2006 to 2010. Can J Public Health.

[33] Wheeler C, Mohle-Boetani J (2019). Completion Rates, Adverse Effects, and Costs of a 3-Month and 9-Month Treatment Regimen for Latent Tuberculosis Infection in California Inmates, 2011-2014. Public Health Reports.

[34] Jafri SM, Singal AG, Kaul D, Fontana RJ (2011). Detection and management of latent tuberculosis in liver transplant patients. Liver Transplantation.

[35] Swift MD, Molella RG, Vaughn AI, Breeher LE, Newcomb RD, Abdellatif S (2020). Determinants of latent tuberculosis treatment acceptance and completion in healthcare personnel. Clin Infect Dis.

[36] Scholten JN, Driver CR, Munsiff SS, Kaye K, Rubino MA, Gourevitch MN (2003). Effectiveness of Isoniazid Treatment for Latent Tuberculosis Infection among Human Immunodeficiency Virus (HIV)-Infected and HIV-Uninfected Injection Drug Users in Methadone Programs. Clin Infect Dis.

[37] Lardizabal A, Passannante M, Kojakali F, Hayden C, Reichman LB (2006). Enhancement of treatment completion for latent tuberculosis infection with 4 months of rifampin. Chest.

[38] Pollock NR, Kashino SS, Napolitano DR, Sloutsky A, Joshi S, Guillet J (2009). Evaluation of the Effect of Treatment of Latent Tuberculosis Infection on QuantiFERON-TB Gold Assay Results. Infect Control Hosp Epidemiol.

[39] Shukla SJ, Warren DK, Woeltje KF, Gruber CA, Fraser VJ (2002). Factors associated with the treatment of latent tuberculosis infection among health-care workers at a midwestern teaching hospital. Chest.

[40] Eastment MC, McClintock AH, McKinney CM, Narita M, Molnar A (2017). Factors that influence treatment completion for latent tuberculosis infection. J Am Board Fam Med.

[41] Page KR, Sifakis F, De Oca RM, Cronin WA, Doherty MC, Federline L (2006). Improved adherence and less toxicity with rifampin vs isoniazid for treatment of latent tuberculosis: A retrospective study. Arch Intern Med.

[42] Macaraig MM, Jalees M, Lam C, Burzynski J (2018). Improved treatment completion with shorter treatment regimens for latent tuberculous infection. Int J Tuberc Lung Dis.

[43] JrCR Horsburgh, Goldberg S, Bethel J, Chen S, Colson PW, Hirsch-Moverman Y (2010). Latent TB infection treatment acceptance and completion in the United States and Canada. Chest.

[44] Plourde PJ, Basham CA, Derksen S, Schultz J, McCulloch S, Larcombe L (2019). Latent tuberculosis treatment completion rates from prescription drug administrative data. Can J Public Health.

[45] Arguello Perez E, Seo SK, Schneider WJ, Eisenstein C, Brown AE (2017). Management of Latent Tuberculosis Infection among Healthcare Workers: 10-Year Experience at a Single Center. Clin Infect Dis.

[46] Medina-Gil C, Dehesa L, Vega A, Kerdel F (2015). Prevalence of latent tuberculosis infection in patients with moderate to severe psoriasis taking biologic therapies in a dermatologic private practice in Miami, Florida. Int J Dermatol.

[47] McNeill L, Allen M, Estrada C, Cook P (2003). Pyrazinamide and rifampin vs isoniazid for the treatment of latent tuberculosis: Improved completion rates but more hepatotoxicity. Chest.

[48] Xu Y, Schwartzman K (2010). Referrals for positive tuberculin tests in new health care workers and students: A retrospective cohort study. BMC Public Health.

[49] Fiske CT, Yan FX, Hirsch-Moverman Y, Sterling TR, Reichler MR (2014). Risk factors for treatment default in close contacts with latent tuberculous infection. Int J Tuberc Lung Dis.

[50] Simkins J, Abbo LM, Camargo JF, Rosa R, Morris MI (2017). Twelve-week rifapentine plus isoniazid versus 9-month isoniazid for the treatment of latent tuberculosis in renal transplant candidates. Transplantation.

[51] Cook PP, Maldonado RA, Yarnell CT, Holbert D (2006). Safety and completion rate of short-course therapy for treatment of latent tuberculosis infection. Clin Infect Dis.

[52] McClintock AH, Eastment M, McKinney CM, Pitney CL, Narita M, Park DR (2017). Treatment completion for latent tuberculosis infection: A retrospective cohort study comparing 9 months of isoniazid, 4 months of rifampin and 3 months of isoniazid and rifapentine. BMC Infect Dis.

[53] Simkins J, Donato-Santana C, Morris MI, Abbo LM, Camargo JF, Anjan S (2020). Treatment of latent tuberculosis infection with short-course regimens in potential living kidney donors. Transpl Infect Dis.

[54] Ronald LA, FitzGerald JM, Bartlett-Esquilant G, Schwartzman K, Benedetti A, Boivin JF (2020). Treatment with isoniazid or rifampin for latent tuberculosis infection: Population-based study of hepatotoxicity, completion and costs. Eur Respir J.

[55] Bourlon C, Camacho-Hernández R, Fierro-Angulo OM, Acosta-Medina AA, Bourlon MT, Niembro-Ortega MD (2020). Latent Tuberculosis in Hematopoietic Stem Cell Transplantation: Diagnostic and Therapeutic Strategies to Prevent Disease Activation in an Endemic Population. Biol Blood Marrow Transplant.

[56] Picone CM, Freitas AC, Gutierrez EB, Avelino-Silva VI (2020). Access and adherence to isoniazid preventive therapy and occurrence of active TB in a cohort of people living with HIV: A retrospective cohort study in Sao Paulo, Brazil. Rev Inst Med Trop Sao Paulo.

[57] Araújo NC, Cruz CM, Arriaga MB, Cubillos-Angulo JM, Rocha MS, Silveira-Mattos PS (2020). Determinants of losses in the latent tuberculosis cascade of care in Brazil: A retrospective cohort study. Int J Infect Dis.

[58] Cataño JC, Morales M (2015). Follow-up results of isoniazid chemoprophylaxis during biological therapy in Colombia. Rheumatol Int.

[59] Joza K, Gallego C, Muñoz L, Poropat A, Salomone C (2019). Incidence of Latent Tuberculosis Infection in a study of household contacts treated in a General Hospital of the City of Buenos Aires. Rev Am med respir.

[60] Stucchi RSB, Boin IFSF, Angerami RN, Zanaga L, Ataide EC, Udo EY (2012). Is isoniazid safe for liver transplant candidates with latent tuberculosis?. Transplant Proc.

[61] Santos DTD, Garcia MC, AANFD C, Pieri FM, Meier DAP, Albanese SPR (2017). Latent tuberculosis infection in persons with HIV/AIDS, associated factors, and progression to active disease in a city in southern Brazil. Cad Saude Publica.

[62] De Lemos AS, Vieira MAMS, Halpern M (2013). Results of implementation of preventive recommendations for tuberculosis after renal transplantation in an endemic area. Am J Transplant.

[63] López G, Wood M, Ayesta FJ (2011). 10 Years of Innovation in the Treatment of Latent Tuberculosis Infection: a Comparison Between Standard and Short Course Therapies in Directly Observed Therapy. Rev Esp Sanid Penit.

[64] Fresard I, Bridevaux PO, Janssens JP (2011). Adverse effects and adherence to treatment of rifampicine 4 months vs isoniazid 6 months for latent tuberculosis: a retrospective analysis. Swiss Med Wkly.

[65] Pina JM, Clotet L, Ferrer A, Sala MR, Garrido P, Salleras L (2013). Cost-effectiveness of rifampin for 4 months and isoniazid for 6 months in the treatment of tuberculosis infection. Respir Med.

[66] Benito N, Sued O, Moreno A, Horcajada JP, González J, Navasa M (2002). Diagnosis and treatment of latent tuberculosis infection in liver transplant recipients in an endemic area. Transplantation.

[67] Sentís A, Vasconcelos P, Machado RS, Caylà JA, Guxens M, Peixoto V (2019). Failure to complete treatment for latent tuberculosis infection and medical-associated factors. Eur J Clin Microbiol Infect Dis.

[68] van Hest R, Baars H, Kik S, van Gerven P, Trompenaars MC, Kalisvaart N (2004). Hepatotoxicity of rifampin-pyrazinamide and isoniazid preventive therapy and tuberculosis treatment. Clin Infect Dis.

[69] Sarivalasis A, Bodenmann P, Langenskiold E, Lutchmaya-Flick C, Daher O (2013). High rate of completion of preventive therapy for latent tuberculosis infection among asylum seekers in a Swiss Canton. Swiss Med Wkly.

[70] Codecasa LR, Murgia N, Ferrarese M, Delmastro M, Repossi AC, Casali L (2013). Isoniazid preventive treatment: Predictors of adverse events and treatment completion. Int J Tuberc Lung Dis.

[71] Villa S, Ferrarese M, Sotgiu G, Castellotti PF, Saderi L, Grecchi C (2019). Latent Tuberculosis Infection Treatment Completion while Shifting Prescription from Isoniazid-Only to Rifampicin-Containing Regimens: A Two-Decade Experience in Milan, Italy. J Clin Med.

[72] Papay P, Primas C, Eser A, Novacek G, Winkler S, Frantal S (2012). Retesting for latent tuberculosis in patients with inflammatory bowel disease treated with TNF-α inhibitors. Aliment Pharmacol Ther.

[73] Abreu C, Afonso J, Camila Dias C, Ruas R, Sarmento A, Magro F (2017). Serial tuberculosis screening in inflammatory bowel disease patients receiving anti-TNFα therapy. J Crohns Colitis.

[74] Anibarro L, Casas S, Paz-Esquete J, Gonzalez L, Pena A, Guerra MR (2010). Treatment completion in latent tuberculosis infection at specialist tuberculosis units in Spain. Int J Tuberc Lung Dis.

[75] Sichletidis L, Settas L, Spyratos D, Chloros D, Patakas D (2006). Tuberculosis in patients receiving anti-TNF agents despite chemoprophylaxis. Int J Tuberc Lung Dis.

[76] Park SJ, Jo KW, Yoo B, Lee CK, Kim YG, Yang SK. (2015). Comparison of LTBI treatment regimens for patients receiving anti-tumour necrosis factor therapy. Int J Tuberc Lung Dis.

[77] Park SH, Lee SJ, Cho YJ, Jeong YY, Kim HC, Lee JD (2016). Prospective Cohort Study of Latent Tuberculosis. Korean J Intern Med.

[78] Park SY, Lee E, Lee EJ, Kim TH, Kim YK (2019). Screening and treatment of latent tuberculosis infection among healthcare workers at a referral hospital in Korea. Infect Chemother.

[79] Lee EH, Kang YA, Leem AY, Park MS, Kim YS, Kim SK (2017). Active Tuberculosis Incidence and Characteristics in Patients Treated with Tumor Necrosis Factor Antagonists According to Latent Tuberculosis Infection. Sci Rep.

[80] Lee CK, Wong SHV, Lui G, Tang W, Tam LS, Ip M (2018). A prospective study to monitor for tuberculosis during anti-tumour necrosis factor therapy in patients with inflammatory bowel disease and immune-mediated inflammatory diseases. J Crohns Colitis.

[81] Noh CS, Kim HI, Choi H, Kim Y, Kim CH, Choi JH (2019). Completion rate of latent tuberculosis infection treatment in patients aged 65 years and older. Respir Med.

[82] Cansu DÜ, Güncan S, Bilge NŞY, Kaşifoğlu T, Korkmaz C (2014). Does isoniazid chemoprophylaxis increase the frequency of hepatotoxicity in patients receiving anti-TNF-α agent with a disease-modifying antirheumatic drug?. Eur J Rheumatol.

[83] Huang YW, Yang SF, Yeh YP, Tsao TCY, Tsao SM (2016). Impacts of 12-dose regimen for latent tuberculosis infection: Treatment completion rate and cost-effectiveness in Taiwan. Medicine.

[84] Huang SF, Chen MH, Wang FD, Tsai CY, Fung CP, Su WJ (2018). Efficacy of isoniazid salvage therapy for latent tuberculosis infection in patients with immune-mediated inflammatory disorders - A retrospective cohort study in Taiwan. J Microbiol Immunol Infect.

[85] Cagatay T, Aydın M, Sunmez S, Cagatay P, Gulbaran Z, Gul A (2010). Follow-up results of 702 patients receiving tumor necrosis factor-alpha antagonists and evaluation of risk of tuberculosis. Rheumatol Int.

[86] Elbek O, Uyar M, Aydın N, Börekçi Ş, Bayram N, Bayram H (2009). Increased risk of tuberculosis in patients treated with antitumor necrosis factor alpha. Clin Rheumatol.

[87] Kyaw NTT, Kumar AMV, Kyaw KWY, Satyanarayana S, Magee MJ, Min AC (2019). IPT in people living with HIV in Myanmar: A five-fold decrease in incidence of TB disease and all-cause mortality. Int J Tuberc Lung Dis.

[88] Almufty HB, Abdulrahman IS, Merza MA (2019). Latent tuberculosis infection among healthcare workers in Duhok province: From screening to prophylactic treatment. Trop Med Infect Dis.

[89] Chee CBE, Teleman MD, Boudville IC, Do SE, Wang YT (2004). Treatment of latent TB infection for close contacts as a complementary TB control strategy in Singapore. Int J Tuberc Lung Dis.

[90] Chee CB, KhinMar KW, Gan SH, Barkham TM, Pushparani M, Wang YT (2007). Latent tuberculosis infection treatment and T-cell responses to Mycobacterium tuberculosis-specific antigens. Am J Respir Crit Care Med.

[91] Khawcharoenporn T, Phetsuksiri B, Rudeeaneksin J, Srisungngam S, Apisarnthanarak A (2017). QuantiFERON-TB Gold In-Tube Test for tuberculosis prevention in HIV-infected patients. Jpn J Infect Dis.

[92] Hanta I, Ozbek S, Kuleci S, Kocabas A (2008). The evaluation of latent tuberculosis in rheumatologic diseases for anti-TNF therapy: Experience with 192 patients. Clin Rheumatol.

[93] Atey TM, Bitew H, Asgedom SW, Endrias A, Berhe DF (2020). Does Isoniazid Preventive Therapy Provide Better Treatment Outcomes in HIV-Infected Individuals in Northern Ethiopia? A Retrospective Cohort Study. AIDS Res Treat.

[94] Johnson JL, Geldenhuys H, Thiel BA, Toefy A, Suliman S, Pienaar B (2014). Effect of isoniazid therapy for latent tb infection on quantiferon-Tb gold in-Tube responses in adults with positive tuberculin skin test results in a high tb incidence area. Chest.

[95] LaCourse SM, Deya RW, Graham SM, Masese LN, Jaoko W, Mandaliya KN (2017). Evaluation of the isoniazid preventive therapy care cascade among HIV-positive female sex workers in Mombasa, Kenya. J Acquir Immune Defic Syndr.

[96] Comstock GW, Ferebee SH, Hammes LM (1967). A controlled trial of community-wide isoniazid prophylaxis in Alaska. Am Rev Respir Dis.

[97] Mount FW, Ferebee SH (1962). The Effect of Isoniazid Prophylaxis on Tuberculosis Morbidity among Household Contacts of Previously Known Cases of Tuberculosis. Am Rev Respir Dis.

[98] Ferebee SH, Mount FW, Murray FJ, Livesay VT (1963). A controlled trial of isoniazid prophylaxis in mental institutions. Nursing Research.

[99] Ferebee SH, Mount FW (1962). Tuberculosis Morbidity in a Controlled Trial of the Prophylactic Use of Isoniazid among Household Contacts. Am Rev Respir Dis.

[100] Dehghani K, Lan Z, Li P, Michelsen SW, Waites S, Benedetti A (2018). Determinants of tuberculosis trends in six Indigenous populations of the USA, Canada, and Greenland from 1960 to 2014: a population-based study. Lancet Public Health.

[101] Geremew D, Geremew H, Tamir M, Adem M, Tegene B, Bayleyegn B (2022). Tuberculosis and isoniazid prophylaxis among adult HIV positive patients on ART in Northwest Ethiopia. PloS one.

[102] Sterling TR, Njie G, Zenner D, Cohn DL, Reves R, Ahmed A (2020). Guidelines for the Treatment of Latent Tuberculosis Infection: Recommendations from the National Tuberculosis Controllers Association and CDC, 2020. MMWR Recomm Rep.

[103] Noreen N, Rakesh J, Sadia L, Tiba R, Hafsa Z, Yook JH (2022). Use of Isoniazid Monotherapy in Comparison to Rifamycin-Based Regimen for the Treatment of Patients With Latent Tuberculosis: A Systematic Review. Cureus.

[104] Bishara H, Ore L, Ravell DW (2014). Compliance with latent tuberculosis treatment: a public health challenge. Harefuah.

[105] Hirsch-Moverman Y, Shrestha-Kuwahara R, Bethel J, Blumberg HM, Venkatappa TK, Horsburgh CR (2015). Latent tuberculous infection in the United States and Canada: who completes treatment and why?. Int J Tuberc Lung Dis.

[106] Liu Q, Abba K, Alejandria MM, Sinclair D, Balanag VM, Lansang MAD (2014). Reminder systems to improve patient adherence to tuberculosis clinic appointments for diagnosis and treatment. Cochrane Database Syst Rev.

[107] Gast A, Mathes T (2019). Medication adherence influencing factors-an (updated) overview of systematic reviews. Systematic reviews.

[108] Lam WY, Fresco P (2015). Medication adherence measures: an overview. Biomed Res Int.

[109] Hayashi PH, Fontana RJ, Chalasani NP, Stolz AA, Talwalkar JA, Navarro VJ (2015). Under-reporting and Poor Adherence to Monitoring Guidelines for Severe Cases of Isoniazid Hepatotoxicity. Clin Gastroenterol Hepatol.

[110] Chung SJ, Byeon S, Choi J (2022). Analysis of Adverse Drug Reactions to First-Line Anti-Tuberculosis Drugs Using the Korea Adverse Event Reporting System. J Korean Med Sci.

[111] Campbell JR, Trajman A, Cook VJ, Johnston JC, Adjobimey M, Ruslami R (2020). Adverse events in adults with latent tuberculosis infection receiving daily rifampicin or isoniazid: post-hoc safety analysis of two randomised controlled trials. Lancet Infect Dis.

[112] Chee CB, Reves R, Zhang Y, Belknap R (2018). Latent tuberculosis infection: Opportunities and challenges. Respirology.

[113] Pieper D, Livia P (2021). Language restrictions in systematic reviews should not be imposed in the search strategy but in the eligibility criteria if necessary. J Clin Epidemiol.

[114] Aronson JK, Ferner RE (2003). Joining the DoTS: New approach to classifying adverse drug reactions. BMJ.

[115] Naranjo CA, Busto U, Sellers EM, Sandor P, Ruiz I, Roberts EA (1981). A method for estimating the probability of adverse drug reactions. Clin Pharmacol Ther.

[116] Houben RM, Menzies NA, Sumner T, Huynh GH, Arinaminpathy N, Goldhaber-Fiebert JD (2016). Feasibility of achieving the 2025 WHO global tuberculosis targets in South Africa, China, and India: a combined analysis of 11 mathematical models. Lancet Glob Health.

