# Supplementary material for: Isoniazid use, effectiveness, and safety for treatment of latent tuberculosis infection: a systematic review
Source: Rev Soc Bras Med Trop. 2024 Mar 25;57:e00402-2024. doi: 10.1590/0037-8682-0504-2023 (PMC10962359; doi:10.1590/0037-8682-0504-2023)
Supplement: Supplementary file 1 [file 1678-9849-rsbmt-57-e00402-2024-supp1.pdf]

Supplementary Table 1 Terms searched for the meditation evidence map

Database	Searched words
Web of Science	TÓPICO: ("Latent Tuberculosis") OR TÓPICO: ("Latent Tuberculoses") OR TÓPICO: ("Tuberculoses, Latent") OR TÓPICO: ("Tuberculosis, Latent") OR TÓPICO: ("Latent Tuberculosis Infection") OR TÓPICO: ("Infection, Latent Tuberculosis") OR TÓPICO: ("Infections, Latent Tuberculosis") OR TÓPICO: ("Latent Tuberculosis Infections") OR TÓPICO: ("Tuberculosis Infection, Latent") OR TÓPICO: ("Tuberculosis Infections, Latent") AND TÓPICO: ("Isoniazid") OR TÓPICO: ("Isonicotinic Acid Hydrazide") OR TÓPICO: ("Hydrazide, Isonicotinic Acid") OR TÓPICO: ("Phthivazide") OR TÓPICO: ("Phthivazid") OR TÓPICO: ("Isonicotinic Acid Vanillylidenehydrazide") OR TÓPICO: ("Acid Vanillylidenehydrazide, Isonicotinic") OR TÓPICO: ("Vanillylidenehydrazide, Isonicotinic Acid") OR TÓPICO: ("Ftivazide") OR TÓPICO: ("Tubazide") OR TÓPICO: ("Isonex")
Scopus	((TITLE-ABS-KEY ("Latent Tuberculosis") OR TITLE-ABS-KEY ("Latent Tuberculoses") OR TITLE-ABS-KEY ("Tuberculoses, Latent") OR TITLE-ABS-KEY ("Tuberculosis, Latent") OR TITLE-ABS-KEY ("Latent Tuberculosis Infection") OR TITLE-ABS-KEY ("Infection, Latent Tuberculosis") OR TITLE-ABS-KEY ("Infections, Latent Tuberculosis") OR TITLE-ABS-KEY ("Latent Tuberculosis Infections") OR TITLE-ABS-KEY ("Tuberculosis Infection, Latent") OR TITLE-ABS-KEY ("Tuberculosis Infections, Latent"))) AND ((TITLE-ABS-KEY ("Isoniazid") OR TITLE-ABS-KEY ("Isonicotinic Acid Hydrazide") OR TITLE-ABS-KEY ("Hydrazide, Isonicotinic Acid") OR TITLE-ABS-KEY ("Phthivazide") OR TITLE-ABS-KEY ("Phthivazid") OR TITLE-ABS-KEY ("Isonicotinic Acid Vanillylidenehydrazide") OR TITLE-ABS-KEY ("Acid Vanillylidenehydrazide, Isonicotinic") OR TITLE-ABS-KEY ("Vanillylidenehydrazide, Isonicotinic Acid") OR TITLE-ABS-KEY ("Ftivazide") OR TITLE-ABS-KEY ("Tubazide") OR TITLE-ABS-KEY ("Isonex")))
Cochrane Library	MeSH descriptor: [Latent Tuberculosis] AND ("Isoniazid"):ti,ab,kw OR ("Isonicotinic Acid Hydrazide"):ti,ab,kw OR ("Hydrazide, Isonicotinic Acid"):ti,ab,kw OR ("Phthivazide"):ti,ab,kw OR ("Phthivazid"):ti,ab,kw" (Word variations have been searched)
PubMed	((((((((((("Isoniazid"[Mesh]) OR "Isoniazid") OR "Isonicotinic Acid Hydrazide") OR "Hydrazide, Isonicotinic Acid") OR "Phthivazide") OR "Phthivazid") OR "Isonicotinic Acid Vanillylidenehydrazide") OR "Acid Vanillylidenehydrazide, Isonicotinic") OR "Vanillylidenehydrazide, Isonicotinic

	Acid") OR "Ftivazide") OR "Tubazide") OR "Isonex")) AND (((((((((("Latent Tuberculosis"[Mesh]) OR "Latent Tuberculosis") OR "Latent Tuberculoses") OR "Tuberculoses, Latent") OR "Tuberculosis, Latent") OR "Latent Tuberculosis Infection") OR "Infection, Latent Tuberculosis") OR "Infections, Latent Tuberculosis") OR "Latent Tuberculosis Infections") OR "Tuberculosis Infection, Latent") OR "Tuberculosis Infections, Latent")
Lilacs	(tw:((tw:("Latent Tuberculosis")) OR (tw:("Latent Tuberculoses")) OR (tw:("Tuberculoses, Latent")) OR (tw:("Tuberculosis, Latent")) OR (tw:("Latent Tuberculosis Infection")) OR (tw:("Infection, Latent Tuberculosis ")) OR (tw:("Infections, Latent Tuberculosis")) OR (tw:("Latent Tuberculosis Infections ")) OR (tw:("Tuberculosis Infection, Latent")) OR (tw:("Tuberculosis Infections, Latent")))) AND (tw:((tw:("Isoniazid")) OR (tw:("Isonicotinic Acid Hydrazide")) OR (tw:("Hydrazide, Isonicotinic Acid")) OR (tw:("Phthivazide")) OR (tw:("Phthivazide")) OR (tw:("Phthivazid")) OR (tw:("Isonicotinic Acid Vanillylidenehydrazide")) OR (tw:("Acid Vanillylidenehydrazide, Isonicotinic")) OR (tw:("Vanillylidenehydrazide, Isonicotinic Acid")) OR (tw:("Ftivazide")) OR (tw:("Tubazide")) OR (tw:("Isonex"))))
EMBASE	'latent tuberculosis' OR 'latent tuberculoses' OR 'tuberculoses, latent' OR 'tuberculosis, latent' OR 'latent tuberculosis infection' OR 'infection, latent tuberculosis' OR 'infections, latent tuberculosis' OR 'latent tuberculosis infections' OR 'tuberculosis infection, latent' OR 'tuberculosis infections, latent' AND ('isoniazid' OR 'isonicotinic acid hydrazide' OR 'hydrazide, isonicotinic acid' OR 'phthivazide' OR 'phthivazid' OR 'isonicotinic acid vanillylidenehydrazide' OR 'acid vanillylidenehydrazide, isonicotinic' OR 'vanillylidenehydrazide, isonicotinic acid' OR 'ftivazide' OR 'tubazide') AND ('isonex'/exp OR 'isonex')
