# Supplementary material for: Isoniazid use, effectiveness, and safety for treatment of latent tuberculosis infection: a systematic review
Source: Rev Soc Bras Med Trop. 2024 Mar 25;57:e00402-2024. doi: 10.1590/0037-8682-0504-2023 (PMC10962359; doi:10.1590/0037-8682-0504-2023)
Supplement: Supplementary file 2 [file 1678-9849-rsbmt-57-e00402-2024-supp2.pdf]

Supplementary Table 2 Characteristics of studies that determine H usage rates for LTBI

Study	Country: study location	Study design (duration)	Sample size and characteristics	Medication
López, Wood, Ayesta [63]	Spain: prison establishment	RC (January 2000 to December 2009)	810 people with indication for LTBI treatment	9H; R + Z; H + R; 4R
Park et al [77]	Korea: hospital	PC (October 2009 to August 2013)	189 active TB contacts	9H; 4R
Lee et al [80]	China: hospital	PC (January 2012 to September 2017)	108 patients with IBD	9H; ID
Young et al [25]	USA: public health clinic	RC (May 2003 to March 2007)	777 patients	9H; 4R
Picone et al [56]	Brazil: HIV clinic	RC (February 2005 to December 2009)	238 HIV positive adult patients	6H
Lee et al [79]	South Korea: hospital	RC (November 2005 to June 2016)	255 patients diagnosed with LTBI before the use of anti-TNF	9H; 3H + R; ID
Li et al [26]	USA: TB clinic	RC (January 2002 to August 2004)	15035 patients started LTBI treatment	H; R
Frésard et al [64]	Switzerland: hospital	RC (January 1993 to December 2007)	624 patients started LTBI treatment	6H or 9H; 4R
Smith et al [27]	Canada: health insurer	RC (January 1998 to December 2003)	9145 patients started LTBI treatment	H; R
Flynn et al [28]	USA: hospital	PC (January 2006 to December 2017)	8479 patients started LTBI treatment	9H; 4R; H + P for 3 months
Park et al [76]	South Korea: hospital	RC (December 2004 to December 2013)	408 IMID patients who started treatment for LTBI	9H; 4R; 3H + R; Steroid, DMARDs, Steroid + DMARDs; ID
Lincoln et al [29]	USA: prison establishment	RC (January 1998 to December 2000)	146 prisoners who started treatment with LTBI	6H; R + Z for 2 months
Sweeney, Ahern, Alston [30]	USA: clinic	RC (2012 to 2015)	164 patients started LTBI treatment	9H; H + P for 3 months
Juarez-Reyes et al [31]	USA: prison establishment	MC (January 2010 to March 2014)	245 prisoners who started treatment with LTBI	9H + pyridoxine; H + P + pyridoxine for 3 months
Noh et al [81]	South Korea: clinic	RC (January 2016 and December 2018)	77 patients started LTBI treatment	9H; 3H + R; 3R
Rivest, Street, Allard [32]	Canada: health insurer	RC (January 2006 to December 2010)	5026 patients started LTBI treatment	9H; 4R
Wheeler, Mohle-Boetani [33]	USA: prison establishment	RC (October 2011 to March 2012)	214 patients with LTBI	9H; 3H + P
Pina et al [65]	Spain: região central de saúde	RC (January 1986 to October 2009)	1002 patients with LTBI	6H + pyridoxine; 4R
Jafri et al [34]	USA: hospital	RC (January 2004 to October 2009)	25 transplant patients with LTB	H + pyridoxine
Araújo et al [57]	Brazil: clinic	RC (2009 to 2014)	475 people with LTBI	6H
Benito et al [66]	Spain: hospital	RC (August 1988 to July 1998)	529 liver transplant patients	12H
Cansu et al [82]	Turkey: departamento de reumatologia	RC (June 2005 to February 2010)	87 patients	9H + pyridoxine
Atey et al [93]	Ethiopia: HIV clinic	RC (2009 to June 30, 2017)	1863 patients	6H
Johnson et al [94]	South Africa: clinic	PC (NR)	78 patients	H
Scholten et al [36]	USA: clínicas de tratamento de dependentes químicos	RC (January 1, 1993 to December 31, 1998)	995 people at high risk for TB	H + pyridoxine; Methadone
Huang et al [84]	Taiwan: clinic	RC (2012 to 2014)	382 pacients	6-9H; Anti-TNF or other biological
Diaz et al [24]	Spain: HIV clinic	PC: (March 2000 to February 2003)	122 patients started LTBI treatment	6-12H; RZ for 2 months; R+ H for 3 months; RHZ for 3 months
Lardizabal et al [37]	USA: clínica de pneumologia	RC: (2000 and 2003)	474 patients	9H; 4R
Pollock et al [38]	USA: clinic	PC: (November 2006 to November 2007)	28 professionals with LTBI	H + pyridoxine
LaCourse et al [95]	Kenya: clinic	RC: (March 2000 to January 2010)	642 female sex workers	H + pyridoxine
Shukla et al [39]	USA: hospital	PC: (January 1, 1994 to May 1, 2000)	404 health professionals	H
Eastment et al [40]	USA: hospital and clinic	RC: (NR)	393 participants	9H; 4R; 3H + R
Cagatay et al [85]	Turkey: department of lung diseases	PC: (August 2005 to July 2008)	702 patients	9H; ID, steroids and MTX
Cataño e Morales [58]	Colombia: infectious diseases clinic	PO (June 2010 to June 2014)	221 patients	9H; 4R; ID
van Hest et al [68]	Netherlands: departamentos de controle de TB	PC e RC (January 2000 to December 2001)	2840 patients	6H; RHZ for 2 months; R+Z for 2 months
Sarivalasis et al [69]	Switzerland: migrant centers	PC (NR)	393 asylum-seekers	9H; 4R
Huang et al [83]	China: hospital	PS (February to July 2014)	691 patients	9H; 3H + P
Page et al [41]	USA: health department	RC (January 1, 1999 to January 31, 2004)	2149 patients	9H; 4R

Supplementary Table 2 Characteristics of studies that determine H usage rates for LTBI

Study	Country: study location	Study design (duration)	Sample size and characteristics	Medication
Macaraig et al [42]	USA: TB clinic	RC (January to June 2015)	449 patients started LTBI treatment	6H; 9H; 4R; 3H + P
Joza et al [59]	Argentina: hospital	RT (January 2016 to February 2018)	340 TB contacts	3H; 6H
Elbek et al [86]	Turkey: NR	PC (December 2005 to December 2007)	240 patients	H; anti-TNF
Kyaw et al [87]	Myanmar: HIV clinic	RC (June 2009 and June 2014)	7177 patients	6H to 9H
Stucchi et al [60]	Brazil: hospital	RC (2008 to 2010)	33 patients with LTBI	6H
Codecasa et al [70]	Italy: hospital	PC (January 1, 1992 to December 31, 2009)	11963 people	6H; R+E
Horsburgh et al [43]	USA e Canada: clinic	RT (2002)	1994 people	6H; 9H; 4R; RZ for 2 months
Bourlon et al [55]	Mexico: hospital	RC (February 2000 to June 2018)	409 patients and donors tested for LTBI	6H
Almufty, Abdulrahman, Merza [88]	Iraq: NR	PT (April to July 2018)	395 healthcare professionals tested for LTBI	6H; 3H + R
Santos et al [61]	Brazil: TB and HIV clinic	DT (2003 to 2014)	690 HIV positive patients with a known result for TT	H
Chee et al [90]	Singapore: Tuberculosis Control Unit	PC (January to September 2005)	226 LTBI positive adolescents and adults	6H; 4R; 4 H + R
Villa et al [71]	Italy: regional TB center	RC (January 1992 to December 2018)	19670 positive people for LTBI	6H; 3H + R; 4R
Plourde et al [44]	Canada: health insurer	RC (April 1, 1999 to March 31, 2014)	5514 people being treated for LTBI	6H; 9H; 4R
Arguello Perez et al [45]	USA: cancer hospital	RC (January 1, 2005 and December 31, 2014)	927 health professionals with LTBI	9H + pyridoxine; 4R; 3H +P
Medina-Gil et al [46]	USA: dermatology private practice center	RC (January 2006 to September 2012)	444 patients with moderate to severe psoriasis receiving biological therapy	9H; RHZE for 6 months
McNeill et al [47]	USA: health department	PC (March 1999 and December 2001)	224 TT positive patients	6H + pyridoxine; Z + R for 2 months
Khawcharoenforn et al [91]	Thailand: HIV clinic	PC (March 1, 2012 to February 29, 2016)	150 HIV patients	9H
Xu, Schwartzman [48]	Canada: respiratory disease center	RC (2006)	630 students and healthcare workers tested with TT	6H; 4R
De Lemos et al [62]	Brazil: NR	RC (December 2004 to July 2010)	535 pacientes que foram consecutivamente submetidos a transplante de rim	6H
Papay et al [72]	Austria: clinic IBD	PC (December 2006 and June 2010)	227 patients with IBD	6-9H
Fiske et al [49]	USA e Canada: health department	PC (January 2002 to December 2006)	1714 contacts diagnosed with LTBI	H; R; H+ P; H+ R; RHZE; Fluoroquinolone
Simkins et al [50]	USA: transplant infectious disease clinic	RC (March 1, 2012 to February 28, 2014)	153 kidney transplant candidates with LTBI	9H + pyridoxine; P for 12 week;
Cook et al [51]	USA: health department	PC (June 2000 to January 2006)	459 patients	9H + pyridoxine; 4R; Z + R for 2 months
Park et al [78]	South Korea: hospital	RC (February to August 2017)	187 positive healthcare professionals for LTBI	9H; 4R; 3H + R
Sentís et al [67]	Portugal: National System of Clinical Surveillance and Monitoring of TB	RC (2013 to 2017)	15478 started treatment for LTBI	6H; 9H; 4R; 3H + R
Abreu et al [73]	Portugal: clinic	PC (July to August 2013)	46 patients with IBD	9H + pyridoxine; infliximab, steroids, azathioprine or MTX
Hanta et al [92]	Turkey: departments of thoracic diseases and rheumatology clinic	PC (April 2005 to January 2008)	192 patients using anti-TNF for rheumatologic diseases	9H + pyridoxine; ID
McClintock et al [52]	USA: clinic	RC (2009 and July 1, 2013 and June 30, 2014)	393 patients with LTBI	9H; 4R, 3H + P
Anibarro et al [74]	Spain: hospital	RC (January 2004 to March 2007)	599 HIV positive who started LTBI treatment	6-9H; 3H + R; 4R; RHZ for 2 months; RHZE for 2 months
Chee et al [89]	Singapore: TB Control unit	NR	895 contacts who started treatment for LTBI	6H; 9H; R
Simkins et al [53]	USA: hospital	RC (January 2009 to December 2017)	40 potential kidney donors treated for LTBI	6H; 9H; 3H + P; 4R
Ronald et al [54]	Canada: NR	RC (2003 to 2007)	10559 patients started LTBI treatment	9H; 4R
Sichletidis et al [75]	Greece: rheumatology center	RC (July 2000 to June 2004)	45 patients using anti-TNF	6H; 3H + R; ID, MTX; Prednisolone

NR not reported, TB tuberculosis, LTBI latent tuberculosis infection, H isoniazid, R Rifampicin, Z pyrazinamide, E ethambutol, P rifapentine, RHZ rifampicin+isoniazid+pyrazinamide, RHZE Rifampicin+Isoniazid+Pyrazinamide+Ethambutol, 3H isoniazid for 3 months, 6H isoniazid for 6 months, 9H isoniazid for 9 months, 4R Rifampicin for 4 months, DT descriptive transversal, PT prospective transversal, RT retrospective transversal, RC retrospective cohort, PC prospective cohort, RO retrospective observational, PO prospective

observational, MC mixed cohort, PS pilot study, IBD Inflammatory Bowel Disease, HIV Human immunodeficiency virus, IMID Immune-mediated inflammatory diseases, DMARDs Disease Activity Modifying Drugs, MTX methotrexate, TNF Tumor Necrosis Factor, TT Tuberculin Test, USA United States of America, ID immunobiological drugs
