# Supplementary material for: Isoniazid use, effectiveness, and safety for treatment of latent tuberculosis infection: a systematic review
Source: Rev Soc Bras Med Trop. 2024 Mar 25;57:e00402-2024. doi: 10.1590/0037-8682-0504-2023 (PMC10962359; doi:10.1590/0037-8682-0504-2023)
Supplement: Supplementary file 3 [file 1678-9849-rsbmt-57-e00402-2024-supp3.pdf]

Supplementary Table 3 Characteristics of prevalence use H and treatment adherence

Study	Prevalence use H n(%)	Treatment adherence n(%)
López, Wood, Ayesta [63]	400 (49.4%)	271 (67.8%) CT
Park et al [77]	22 (11.6%)	9 (40.9%) CT
Lee et al [80]	14 (13.0%)	14 (100%) CT
Young et al [25]	639 (82.2%)	415 (65.0%) CT
Picone et al [56]	210 (88.2%)	196 (93.3%) adherents
Lee et al [79]	219 (89.0%)	186 (84.9%) CT
Li et al [26]	14030 (93.3%)	6187 (44.1%) CT
Frésard et al [64]	426 (68.3%)	316 (74.0%) CT
Smith et al [27]	8686 (95.0%)	4699 (54.1%) CT 6 months
Flynn et al [28]	3902 (46.0%)	NR
Park et al [76]	61 (15.0%)	45 (73.8%) CT
Lincoln et al [29]	69 (47.0%)	51 (74.0%) CT
Sweeney, Ahern, Alston [30]	82 (50.0%)	61 (74.4%) CT
Juarez-Reyes et al [31]	154 (62.8%)	28 (18.0%) CT
Noh et al [81]	12 (15.6%)	9 (75.0%) CT
Rivest, Street, Allard [32]	2895 (57.6%)	907 (31.3%) get ≥270 doses; 1625 (56.1%) get ≥180 doses
Wheeler, Mohle-Boetani [33]	92 (43.0%)	39 (42.0%) CT
Pina et al [65]	863 (86.1%)	648 (75.1%; IC 95%: 73.1–78.0) CT
Jafri et al [34]	15 (60.0%)	7 (46.0%) CT 6 months
Swift et al [35]	173 (89,6%)	118 (68%) CT
Araújo et al [57]	300 (85.5%)	249 (83.0%) CT
Benito et al [66]	23 (4.4%)	NR
Cansu et al [82]	61 (70.1%)	46 (75.6%) CT
Atey et al [93]	621 (33.3%)	NR
Johnson et al [94]	39 (50.0%)	35 (90.0%) received 180 doses over the 7-month period
Scholten et al [36]	607 (61.0%)	259 (26.1%) have completed 6 or more months of treatment
Huang et al [84]	50 (13.1%)	50 (100%) received for 6-9 months (average of 7 months)
Diaz et al [24]	Total: 105 (86.0%); 6H: 51 (41.8%); 9H: 26 (21.3%); 12H: 22 (18.0%)	29 (56.9%) CT 6 months; 14 (53.8%) CT 9 months; 8 (36.4%) CT 12 months
Lardizabal et al [37]	213 (44.9%)	113 (53.2%) CT
Pollock et al [38]	17 (60.7%)	5 (29.4%) CT
LaCourse et al [95]	351 (54.7%)	249 (71.0%) CT 6 months
Shukla et al [39]	396 (98.0%)	318/388 (82.0%) were compliant with therapy; 62 (16.0%) CT 6 months
Eastment et al [40]	222 (56.5%)	115 (44.4%) CT
Cagatay et al [85]	583 (83.0%)	NR
Cataño e Morales [58]	218 (98.6%)	186 (84.2%) CT 9 months
van Hest et al [68]	528 (18.6%)	NR
Sarivalasis et al [69]	1 (0.3%)	NR
Huang et al [83]	590 (85.4%)	515 (87.3%)
Page et al [41]	770 (35.8%)	405 (52.6%) CT
Macaraig et al [42]	55 (12.0%)	27 (49.0%) CT
Joza et al [59]	55 (16.2%)	41 (75.0%)
Elbek et al [86]	185 (77.1%)	NR
Kyaw et al [87]	1278 (18%)	855 (67.0%) CT 6 or 9 months
Stucchi et al [60]	27 (81.8%)	18 (66.6%) RM for 6 months; 8 (29.6%) RM for 2 to 4 months
Codecasa et al [70]	Total: 11832 (98.0%)	8764 (74.1%) CT
Horsburgh et al [43]	6H: 181 (9.1%); 9H: 1674 (84.0%)	9H: 1307 (78.1%) completed 6 months but failed to complete the final 3 months
Bourlon et al [55]	78 (72.9%)	78 (100%) have completed at least 6 months of treatment
Almufty, Abdulrahman, Merza [88]	12 (38.7%)	12 (100%)
Santos et al [61]	39 (59.1%)	26 (66.6%)
Chee et al [90]	216 (96.0%)	216 (100%)
Villa et al [71]	15605 (79.3%)	12141 (77.8%) CT
Plourde et al [44]	4985 (90.4%)	63.8% for 180 days; 40.4% for 270 days
Arguello Perez et al [45]	202 (56.0%)	117 (58.0%) CT 9 months; 20 (10.0%) CT 6-8.5 months
Medina-Gil et al [46]	29 (6.5%)	29 (100%)
McNeill et al [47]	114 (50.9%)	67 (59.0%) CT
Khawcharoenforn et al [91]	16 (10.7%)	15 (75.0%) CT; 1 (5.0%) RM for 6 months
Xu, Schwartzman [48]	124 (19.6%)	74 (60.0%) CT; 2 changed from R to H and CT
De Lemos et al [62]	274 (51.0%)	59 de 65 (91.0%) CT
Papay et al [72]	32 (17.4%)	32 (100%)
Fiske et al [49]	807 (70.0%)	456 (57.0%) CT
Simkins et al [50]	110 (72.0%)	52 (47.0%) CT

Supplementary Table 3 Characteristics of prevalence use H and treatment adherence

Study	Prevalence use H n(%)	Treatment adherence n(%)
Cook et al [51]	149 (32.5%)	98 (65.8%) CT
Park et al [78]	43 (31.4%)	34 (81.0%) CT
Sentís et al [67]	6H: 7332 (54.9%); 9H: 4298 (32.2%); <4H: 263 (2.0%)	11893 (89.1%) CT
Abreu et al [73]	15 (93.0%)	14 (93.0%) CT
Hanta et al [92]	129 (67.2%)	NR
McClintock et al [52]	224 (57.0%)	115 (51.8%) CT
Anibarro et al [74]	6H: 466 (77.8%); 9H: 80 (13.4%)	NR
Chee et al [89]	876 (98.0%)	NR
Simkins et al [53]	6H: 1 (4.0%); 9H: 3 (13.0%)	NR
Ronald et al [54]	9684 (93.0%)	3573 (36.9%) CT
Sichletidis et al [75]	40 (88.9%)	NR

NR not reported, H isoniazid, 3H isoniazid for 3 months, 6H isoniazid for 6 months, 9H isoniazid for 9 months, CT completed the treatment, RM received medication
