# Supplementary material for: Isoniazid use, effectiveness, and safety for treatment of latent tuberculosis infection: a systematic review
Source: Rev Soc Bras Med Trop. 2024 Mar 25;57:e00402-2024. doi: 10.1590/0037-8682-0504-2023 (PMC10962359; doi:10.1590/0037-8682-0504-2023)
Supplement: Supplementary file 4 [file 1678-9849-rsbmt-57-e00402-2024-supp4.pdf]

Supplementary Table 4 Characteristics of adverse events to isoniazid

Study	AEI(s)n(%)
López, Wood, Ayesta [63]	37 (9.2%) total AE; 9 (2.2%) gastric intolerance; 8 (2.0%) malaise; 14 (3.5%) increased AST/ALT; 2 (0.5%) non-specific discomfort; 1 (0.2%) heart attack and icterus; 1 (0.2%) psychotic break
Young et al [25]	29 (4.5%) neuropathy; 14 (2.2%) symptomatic elevation in ALT level > 2.5 times the upper limit of normal (⩾100 IU/L); 6 (1.0%) symptomatic elevation in ALT level > 5 times the upper limit of normal (⩾200 U/L);
Picone et al [56]	1 (0.5%) GI event
Lee et al [79]	9 (4.1%) stopped using the drug due to side effects
Li et al [26]	154 (1.1%) patients reported adverse reactions to treatment
Frésard et al [64]	43 (10.1%) total; 26 (6.1%) total hepatotoxicity; 7 (3.5%) AST/ALT ≥3 - <5 times the upper limit of normal; 19 (4.5 %) AST/ALT ≥5 times the upper limit of normal; 6 (1.4%) clinical hepatitis; 17 (4.0%) other reactions; 3 (0.7%) skin reactions; 5 (1.2%) GI; 6 (1.4%) neurological; 3 (0.7%) asthenia
Park et al [76]	7 (11.5%) total; 1 (1.6%) GI; 6 (9.8%) hepatotoxicity, light: 4 (6.6%), moderate to severe: 2 (3.3%)
Sweeney, Ahern, Alston [30]	100 (1.2%) adverse reactions for person
Juarez-Reyes et al [31]	1 (0.6%) people stop treatment due to hepatotoxicity
Noh et al [81]	3 (25.0%) AE; 1 (8.3%) itching; 2 (16.7%) increased AST/ALT; 2 (16.7%) hepatotoxicity (grade 1)
Wheeler, Mohle-Boetani [33]	13 (14.1%) hepatotoxicity
Pina et al [65]	40 (4.6%) people who did not demand suspension; 51 (6.0%) people treatment suspended; 3 (0.3%; IC 95%: 0.1-1.0) digestive intolerance; 2 (0.2%; IC 95%: 0.0–0.8) peripheral polyneuritis; 35 (4.1%; IC 95%: 2.7–5.1) asymptomatic elevated ALT levels; 5 (0.6%; IC 95%: 0.2–1.3) hypersensitivity; 46 (5.3%; IC 95%: 3,7–6.9) Hepatitis
Jafri et al [34]	3 (20.0%) thrombocytopenia; 1 (6.7%) nausea and vomiting; 1 (6.7%) slight rejection; 1 (6.7%) hepatotoxicity; 1 (6.7%) tacrolimus high depressions
Araújo et al [57]	AE frequency was 2.0%
Benito et al [66]	11 (48.0%) changes in liver function
Cansu et al [82]	7 (11.0%) increased AST/ALT
Diaz et al [24]	3 (2.8%) hepatotoxicity; 1 (0.9%) rash; 1 (0.9%) gastric intolerance
Lardizabal et al [37]	5 (2.4%) rash/allergy; 2 (0.9%) headache/flu symptoms; 3 (1.4%) hepatitis; 3 (1.4%) abdominal discomfort
LaCourse et al [95]	16 (5.0%) drug intolerance
Eastment et al [40]	58 (26.1%) total; 9 (4.0%) nausea/vomiting; 15 (6.7%) abdominal discomfort/pain - 8 (3.6%) burning/neuropathic pain; 10 (4.5%) rash; 33 (14.9%) other
Cataño e Morales [58]	32 (17.2%) intolerance or toxicity (allergic reaction, gastric intolerance and hepatotoxicity)
van Hest et al [68]	18 (3.4%) hepatotoxicity
Huang et al [83]	28 (4.7%) not CT due to AE; 21 (3.6%) increased AST/ALT grade 1 to 4
Page et al [41]	76 (11.3%) clinically recognized adverse reactions; 31 (4.6%) permanent discontinuations of treatment; 12 (1.8%) hepatotoxicity; 7 (1.0%) increased AST/ALT; 19 (2.8%) GI events; 14 (2.1%) itching; 7 (1.0%) fatigue; 17 (2.5%) others
Macaraig et al [42]	2 (4.0%) discontinued treatment due to AE
Elbek et al [86]	1 (0.5%) hepatotoxicity
Stucchi et al [60]	No patient developed clinical decompensation or laboratory abnormalities
Bourlon et al [55]	No case of H-related toxicity that warranted drug discontinuation
Almufty, Abdulrahman, Merza [88]	2 (6.5%) received alternative therapy for drug intolerance
Villa et al [71]	1954 (12.8%) EAs; 833 (5.5%) elevated transaminase: 56 (0.4%) severe hepatitis, 342 (2.2%) GI problems, 383 (2.5%) Central Nervous System Problems, 125 (0.8%) peripheral neuropathy, 60 (0.4%) dermatological events
Arguello Perez et al [45]	82 (41.0%) total; 14 (7.0%) more than 1 side effect; 2 (1.0%) urticaria; 5 (3.0%) fatigue/weakness; 9 (4.0%) GI symptoms; 3 (1.0%) increased total bilirubin; 57 (28.0%) increased AST/ALT; 7 (1.0%) increased AST/ALT grade 3 and 4 leading to discontinuation of treatment; 1 (1.0%) increased alkaline phosphatase; 3 (1.0%) itching; 2 (1.0%) Neuropathy
McNeill et al [47]	5 (4.0%) hepatotoxicity
Khawcharoenforn et al [91]	1 (5.0%) Asymptomatic elevation of AST/ALT
De Lemos et al [62]	1 patient discontinued treatment with H due to AE, peripheral neuropathy and GI intolerance
Simkins et al [50]	12 (11.0%) discontinuation due to EA; 6 (5.0%): increase 2 times AST/ALT (5 discontinued treatment); 2 (2.0%) neuropathy; 2 (2.0%) hepatomegaly; 1 (1.0%) dizziness; 1 (1.0%) psychosis
Cook et al [51]	17 (11.4%) hepatotoxicity
Park et al [78]	Nº de EAs - 1: 17 (39.5%), 2: 7 (16.3%), 3: 1 (2.3%); 17 (39.5%) hepatotoxicity
Abreu et al [73]	1 (6%) GI intolerance
Chee et al [89]	4 (0.5%) drug induced hepatitis; 11 (1.3%) increases liver enzymes <3 times; 11 (1.3%) rash; 4 (0.5%) headache; 2 (0.2%) itching; 2 (0.2%) Nausea/loss of appetite; 1 (0.1%) Somnolence, 1 (0.1%) Reduced color vision, 1 (0.1%) periorbital edema, 1 (0.1%) xerostomia
Ronald et al [54]	15 (0.2%) hepatic EA: 1 died, 2 received liver transplant

AE(s) adverse event, AEI(s) adverse events to isoniazid, GI gastrointestinal, AST aspartate aminotransferase, ALT alanine aminotransferase, CT completed the treatment, RM received medication
