# Supplementary material for: Isoniazid use, effectiveness, and safety for treatment of latent tuberculosis infection: a systematic review
Source: Rev Soc Bras Med Trop. 2024 Mar 25;57:e00402-2024. doi: 10.1590/0037-8682-0504-2023 (PMC10962359; doi:10.1590/0037-8682-0504-2023)
Supplement: Supplementary file 5 [file 1678-9849-rsbmt-57-e00402-2024-supp5.pdf]

Supplementary Table 5 Characteristics of treatment effectiveness with isoniazid

Study	Effectiveness n(%)
López, Wood, Ayesta [63]	1 (0.3%) abandoned treatment for developing active TB
Lee et al [80]	1 (7.1%) patient with Crohn's disease and LTBI subsequently developed active TB despite completion of treatment
Picone et al [56]	3 (1.5%) of adherents (95% confidence interval 0.3-4.4%) developed active TB after a mean follow-up of 114 months
Lee et al [79]	5 (2.3%) developed active TB within 6 months of starting anti-TNF therapy
Flynn et al [28]	10 (0.3%) patients who developed active TB
Park et al [76]	2 (3.3%) developed active TB
Sweeney, Ahern, Alston [30]	No active TB cases
Jafri et al [34]	None of the patients had TB reactivation during a follow-up of an average of 34 months
Araújo et al [57]	2 people developed active TB during follow-up
Benito et al [66]	None developed post-transplant TB
Atey et al [93]	28 (4.5%) patients developed TB
Johnson et al [94]	No patient developed TB during the 22.5 months of follow-up
Scholten et al [36]	1 (0.2%) HIV positive participant developed TB after completing 8 months of treatment
Huang et al [84]	No patient had recurrence of active TB
Lardizabal et al [37]	1 (0.5%) active TB diagnosis
Cagatay et al [85]	5 (0,8%) patients developed active TB
Cataño e Morales [58]	7 (3.2%) patients developed active TB, ranging from 2 to 12 months after starting biological therapy
Huang et al [83]	2 (0.3%) developed active TB during treatment
Page et al [41]	No patient using H developed TB
Elbek et al [86]	2 (1.1%) patients developed TB
Kyaw et al [87]	16 (2.0%) patients who completed treatment developed TB
Stucchi et al [60]	No patient developed TB
Bourlon et al [55]	There were no cases of active TB up to one year after transplantation
Almufty, Abdulrahman, Merza [88]	No cases of active TB were found in healthcare workers with LTBI
Santos et al [61]	13 (19.7%) individuals diagnosed with LTBI progressed to active TB
Villa et al [71]	6 people develop TB during preventive treatment; 57 (0.4%) patients who completed or interrupted preventive treatment were diagnosed with TB
Medina-Gil et al [46]	No evidence of active TB in treated cases; 1 patient had pulmonary TB during the study and was treated with the protocol RHZE
Khawcharoenforn et al [91]	None of the LTBI patients who completed at least 6 months of treatment developed active TB during the 3-year follow-up
Xu, Schwartzman [48]	No health worker or student had active TB
De Lemos et al [62]	2 (1.0%) pacientes tiveram TB ativa durante o acompanhamento
Papay et al [72]	No cases of TB were detected in the study
Fiske et al [49]	14 patients developed TB during follow-up, 9 used H for less than 6 months and 2 for 6 or more months
Simkins et al [50]	No cases of TB reactivation after transplantation
Abreu et al [73]	No cases of active TB were reported
Hanta et al [92]	3 (1.6%) patients developed active TB, but none of them had used H
Chee et al [89]	1 (0.1%) developed TB, however it was a patient who received 6 months when he should have received 9
Simkins et al [53]	No cases of active TB were reported
Sichletidis et al [75]	4 (10.0%) patients developed active TB 2-35 months after starting anti-TNF therapy

NR not reported, TB tuberculosis, LTBI latent tuberculosis infection, H isoniazid, HIV Human immunodeficiency virus, TNF Tumor Necrosis Factor
