# Supplementary material for: Isoniazid use, effectiveness, and safety for treatment of latent tuberculosis infection: a systematic review
Source: Rev Soc Bras Med Trop. 2024 Mar 25;57:e00402-2024. doi: 10.1590/0037-8682-0504-2023 (PMC10962359; doi:10.1590/0037-8682-0504-2023)
Supplement: Supplementary file 6 [file 1678-9849-rsbmt-57-e00402-2024-supp6.pdf]

Supplementary Table 6. Quality assessment of cross-sectional studies using the *Quality Assessment Tool for Observational Cohort and Cross-Sectional Studies*

Criteria	López, Wood, Ayesta [63]	Park et al [77]	Lee et al [80]	Young et al [25]	Picone et al [56]	Lee et al [79]	Li et al [26]	Frésard et al [64]	Smith et al [27]	Flynn et al [28]	Park et al [76]	Lincoln et al [29]	Sweeney, Ahern, Alston [30]	Juarez-Reyes et al [31]	Noh et al [81]	Rivest, Street, Allard [32]	Wheeler, Mohle-Boetani [33]	Pina et al [65]	Jafri et al [34]	Swift et al [35]
Question	Y	Y	Y	Y	Y	Y	Y	Y	Y	N	Y	Y	Y	Y	Y	Y	Y	Y	Y	Y
Population	Y	Y	Y	Y	Y	Y	Y	Y	Y	Y	Y	Y	Y	N	Y	Y	Y	Y	Y	Y
Participation Rate	Y	Y	Y	Y	Y	Y	Y	Y	Y	Y	Y	Y	NR	N	Y	Y	Y	Y	Y	Y
Recruitment	Y	Y	Y	Y	Y	Y	Y	Y	Y	Y	Y	Y	NR	Y	Y	Y	Y	Y	Y	Y
Sample size	N	NR	NR	NR	NR	NR	Y	N	Y	N	N	N	N	N	NR	NR	NR	N	N	N
Exposure and outcome	Y	Y	Y	Y	Y	Y	Y	Y	Y	N	Y	Y	N	Y	Y	Y	Y	Y	Y	Y
Timeframe between exposure and outcome	Y	Y	Y	Y	Y	Y	Y	Y	Y	Y	Y	Y	NR	Y	Y	Y	Y	Y	Y	Y
Levels of exposure	N	Y	Y	Y	N	N	Y	Y	Y	N	N	N	N	N	Y	N	N	NA	N	Y
Exposure measure	Y	Y	Y	Y	Y	Y	Y	Y	Y	N	Y	Y	NR	N	Y	Y	Y	Y	Y	Y
Repeated exposure measurement	Y	Y	Y	Y	Y	Y	Y	Y	Y	N	NR	Y	N	N	Y	N	N	Y	Y	N
Outcome measure	Y	Y	Y	Y	Y	Y	Y	Y	Y	NR	Y	Y	N	N	Y	NR	Y	Y	Y	Y
Blinding of assessors	NR	NR	NR	NR	NR	NR	NR	NR	NR	NR	NR	NR	NR	N	NR	NR	NR	NR	NR	NR
Follow-up rate	Y	Y	Y	Y	N	Y	Y	Y	Y	Y	Y	Y	NR	N	Y	Y	Y	Y	Y	Y
Statistical analyses	NR	Y	Y	Y	NR	Y	Y	Y	Y	Y	NR	N	NR	N	Y	Y	NR	N	Y	NR
Overall quality	Fair	Good	Good	Good	Fair	Good	Good	Good	Good	Good	Fair	Fair	Fair	Fair	Good	Good	Fair	Fair	Good	Fair

Rater #1 initials and # 2: BMCSA e MMT; CD, cannot determine; NA, not applicable; NR, not reported; Y, yes; N, no

Continuation Supplementary Table 6. Quality assessment of cross-sectional studies using the *Quality Assessment Tool for Observational Cohort and Cross-Sectional Studies*

Criteria	Araújo et al [57]	Benito et al [66]	Cansu et al [82]	Atey et al [93]	Johnson et al [94]	Scholten et al [36]	Huang et al [84]	Diaz et al [24]	Lardizabal et al [37]	Pollock et al [38]	LaCourse et al [95]	Shukla et al [39]	Eastment et al [40]	Sentís et al [67]	Cagatay et al [85]	Cataño e Morales [58]	van Hest et al [68]	Sarivalasis et al [69]	Huang et al [83]	Page et al [41]
Question	Y	Y	Y	Y	Y	Y	Y	Y	N	Y	Y	Y	Y	Y	Y	Y	Y	Y	Y	Y
Population	Y	Y	Y	Y	Y	Y	Y	Y	Y	Y	Y	Y	Y	Y	Y	Y	Y	Y	Y	Y
Participation Rate	N	Y	Y	Y	Y	Y	Y	Y	Y	N	Y	Y	NA	NA	Y	Y	Y	Y	Y	Y
Recruitment	Y	Y	NR	Y	Y	Y	Y	Y	Y	Y	Y	Y	Y	Y	Y	Y	Y	Y	Y	Y
Sample size	N	NR	NR	Y	N	NR	NR	NR	NR	NR	Y	Y	NA	Y	N	N	Y	N	N	Y
Exposure and outcome	Y	Y	Y	Y	Y	Y	Y	Y	Y	Y	Y	Y	NA	NA	Y	Y	Y	Y	Y	Y
Timeframe between exposure and outcome	Y	Y	Y	Y	NR	Y	NR	Y	Y	Y	Y	Y	NA	NA	Y	Y	Y	Y	Y	Y
Levels of exposure	Y	Y	Y	NR	N	NR	Y	NR	NA	NR	N	Y	NA	NA	NA	NA	NA	NA	NA	NA
Exposure measure	Y	Y	Y	Y	Y	Y	Y	Y	Y	Y	NR	NA	NA	NA	NA	Y	Y	Y	Y	Y
Repeated exposure measurement	N	N	N	Y	N	NR	N	NR	N	Y	NR	Y	NA	NA	NA	NA	NA	Y	Y	Y
Outcome measure	Y	Y	Y	Y	Y	Y	Y	Y	NR	N	N	Y	NA	NA	NA	Y	Y	Y	Y	Y
Blinding of assessors	NR	NR	N	NR	NR	NR	NR	NR	N	NR	NA	NA	NA	NA	NA	NR	NR	NR	NR	NR
Follow-up rate	Y	Y	NR	Y	Y	Y	Y	Y	Y	N	Y	Y	NA	NA	N	NA	Y	N	Y	Y
Statistical analyses	N	N	N	N	N	NR	Y	Y	NR	NR	N	Y	NA	NA	NA	N	Y	N	Y	Y
Overall quality	Fair	Good	Fair	G	Fair	Fair	Fair	Fair	Fair	Fair	Fair	Good	Fair	G	Fair	Good	Fair	Fair	Good	Good

Rater #1 initials and # 2: BMCSA e MMT; CD, cannot determine; NA, not applicable; NR, not reported; Y, yes; N, no

Continuation Supplementary Table 6. Quality assessment of cross-sectional studies using the *Quality Assessment Tool for Observational Cohort and Cross-Sectional Studies*

Criteria	Macaraig et al [42]	Joza et al [59]	Elbek et al [86]	Kyaw et al [87]	Stucchi et al [60]	Codecasa et al [70]	Horsburgh et al [43]	Bourlon et al [55]	Almufty, Abdulrahman, Merza [88]	Santos et al [61]	Chee et al [90]	Villa et al [71]	Plourde et al [44]	Arguello Perez et al [45]	Noh et al., 2019 [81]	Medina-Gil et al [46]	McNeill et al [47]	Khawcharoenporn et al [91]	Xu, Schwartzman [48]	De Lemos et al [62]
Question	Y	Y	Y	Y	Y	Y	Y	Y	Y	Y	Y	Y	Y	Y	Y	Y	Y	Y	Y	Y
Population	Y	Y	Y	Y	Y	Y	Y	Y	Y	Y	Y	Y	Y	Y	Y	Y	Y	Y	Y	Y
Participation Rate	Y	Y	Y	Y	Y	Y	Y	Y	Y	Y	Y	Y	Y	Y	Y	Y	Y	Y	Y	Y
Recruitment	Y	Y	Y	Y	Y	Y	Y	Y	Y	Y	Y	Y	Y	Y	Y	Y	Y	Y	Y	Y
Sample size	Y	N	N	Y	N	Y	Y	N	Y	N	N	N	N	N	NR	N	N	N	N	N
Exposure and outcome	Y	Y	Y	Y	Y	Y	Y	Y	Y	Y	Y	Y	Y	Y	Y	Y	Y	Y	Y	Y
Timeframe between exposure and outcome	Y	Y	Y	Y	Y	Y	Y	Y	Y	Y	Y	Y	Y	Y	Y	Y	Y	Y	Y	Y
Levels of exposure	NA	NA	NA	NA	NA	NA	NA	NA	NA	NA	NA	NA	NA	NA	Y	NA	NA	NA	NA	NA
Exposure measure	Y	Y	Y	Y	Y	Y	Y	Y	Y	Y	Y	Y	Y	Y	Y	Y	Y	Y	Y	Y
Repeated exposure measurement	Y	Y	Y	Y	Y	Y	Y	Y	N	Y	N	Y	Y	Y	Y	Y	Y	Y	Y	Y
Outcome measure	Y	Y	Y	Y	Y	Y	Y	N	Y	Y	Y	Y	Y	Y	Y	Y	Y	Y	Y	Y
Blinding of assessors	NR	NR	NR	NR	NR	NR	NR	NR	NR	NR	NR	NR	NR	NR	NR	NR	NR	NR	NR	NR
Follow-up rate	Y	Y	Y	Y	Y	Y	Y	Y	NA	N	Y	Y	Y	N	Y	Y	Y	N	Y	Y
Statistical analyses	Y	N	N	Y	N	Y	Y	N	Y	Y	N	N	Y	N	Y	N	N	N	N	N
Overall quality	Good	Fair	Fair	Good	Fair	Good	Good	Fair	Good	Good	Fair	Fair	Good	Fair	Good	Good	Good	Fair	Fair	Good

Rater #1 initials and # 2: BMCSA e MMT; CD, cannot determine; NA, not applicable; NR, not reported; Y, yes; N, no

Continuation Supplementary Table 6. Quality assessment of cross-sectional studies using the *Quality Assessment Tool for Observational Cohort and Cross-Sectional Studies*

Criteria	Papay et al [72]	Fiske et al [49]	Simkins et al [50]	Cook et al [51]	Park et al [78]	Abreu et al [73]	Hanta et al [92]	McClintock et al [52]	Anibarro et al [74]	Chee et al [89]	Simkins et al [53]	Ronald et al [54]	Sichletidis et al [75]
Question	Y	Y	Y	Y	Y	Y	Y	Y	Y	Y	Y	Y	Y
Population	Y	Y	Y	Y	Y	Y	Y	Y	Y	Y	Y	Y	Y
Participation Rate	Y	Y	Y	Y	Y	Y	Y	Y	Y	Y	Y	Y	Y
Recruitment	Y	Y	Y	Y	Y	Y	Y	Y	Y	Y	Y	Y	Y
Sample size	N	N	N	N	N	N	N	Y	Y	N	N	N	N
Exposure and outcome	Y	Y	Y	Y	Y	Y	Y	Y	Y	Y	Y	Y	Y
Timeframe between exposure and outcome	Y	Y	Y	Y	Y	Y	Y	Y	Y	Y	Y	Y	Y
Levels of exposure	NA	NA	NA	NA	NA	NA	NA	NA	NA	NA	NA	NA	NA
Exposure measure	Y	Y	Y	Y	Y	Y	Y	Y	Y	Y	Y	Y	Y
Repeated exposure measurement	Y	Y	Y	Y	Y	Y	Y	Y	Y	Y	Y	Y	Y
Outcome measure	Y	Y	Y	Y	Y	Y	Y	Y	Y	Y	Y	Y	Y
Blinding of assessors	NR	NR	NR	NR	NR	NR	NR	NR	NR	NR	NR	NR	NR
Follow-up rate	Y	Y	Y	Y	Y	Y	N	Y	Y	Y	Y	Y	Y
Statistical analyses	N	Y	Y	N	N	N	N	Y	Y	N	N	Y	N
Overall quality	Fair	Good	Good	Fair	Fair	Fair	Fair	Good	Good	Fair	Fair	Good	Fair

Rater #1 initials and # 2: BMCSA e MMT; CD, cannot determine; NA, not applicable; NR, not reported; Y, yes; N, no
